# LINC00511/hsa-miR-573 axis-mediated high expression of Gasdermin C associates with dismal prognosis and tumor immune infiltration of breast cancer

**DOI:** 10.1038/s41598-022-19247-9

**Published:** 2022-08-30

**Authors:** Kai Sun, Ri-xin Chen, Jing-zhang Li, Zhan-xiong Luo

**Affiliations:** Department of Oncology, Liuzhou People’s Hospital, Liuzhou, 545001 Guangxi Zhuang Autonomous Region China

**Keywords:** Cancer, Computational biology and bioinformatics, Immunology

## Abstract

Breast cancer (BC) is considered the second commonest human carcinoma and the most incident and mortal in the female population. Despite promising treatments for breast cancer, mortality rates of metastatic disease remain high. Gasdermin C (GSDMC) is an affiliate of the gasdermin (GSDM) family, which is involved in the process of pyroptosis. Pyroptosis is implicated in tumorigenesis, but the role of GSDMC in cancer cells is yet to be fully elucidated. In this study, we investigated the role and mechanism of GSDMC in breast cancer. We conducted a pan-cancer analysis of the expression and prognosis of GSDMC utilizing multidimensional data from The Cancer Genome Atlas (TCGA). We investigated GSDMC expression levels in 15 BC tissues and matched adjacent normal tissues by immunohistochemistry (IHC). Further verification was performed in the Gene Expression Omnibus (GEO) database. We discovered that elevated GSDMC expression was considerably linked to a worse prognosis in breast invasive carcinoma (BRCA). Next, we identified noncoding RNAs (ncRNAs) which contributing to higher expression of GSDMC by a series of expression, survival, and correlation analysis. We finally identified LINC00511/hsa-miR-573 axis to be the most promising ncRNA-associated pathways that account for GSDMC in BRCA. Furthermore, we demonstrated the significant correlations between GSDMC expression and immune infiltrates, immune checkpoints, and immune markers in BRCA. This study illustrated that ncRNAs-mediated upregulation of GSDMC linked to dismal prognosis and also exhibited a correlation with tumor immune cell infiltration in BRCA. It is anticipated to offer novel ideas for the link between pyroptosis and tumor immunotherapy.

## Introduction

Breast cancer is considered the commonest type of neoplasia in women worldwide and the second most incident and mortal in the human population^1–3^. Surgery, radiation therapy, chemotherapeutics, and endocrine therapy are traditional treatments for BC^4–7^. In recent years, molecular phenotyping, genotyping alters the landscape of BC treatment. The treatment of BC is entering a new era of targeted therapy and immunotherapy^8–10^.

Cell death is an extremely complicated and essential self-destruction biological process that performs the function of maintaining homeostatic balance^11,12^. In vivo, cell death includes apoptosis, pyroptosis, oncosis, and autophagy^13,14^. Pyroptosis, a form of programmed necrotic cell death, is essential for enhancing the defense of the host against danger signals and infections^12,15^. Nevertheless, extreme pyroptosis could also contribute to immunological diseases and septic shock^11,16^. Pyroptosis is a current research hotspot that involves various diseases and conditions, such as chronic inflammation, cardiovascular diseases, bacterial infections, neurodegenerative diseases, autoimmune diseases, and tumors^12,15,17^.

The Gasdermin family of proteins are the most important regulators of pyroptosis^18–20^. 6 members make up the GSDM family in humans and include: GSDMA, GSDMB, GSDMC, GSDMC, GSDME (DFNA5), and GSDMF (DFNB59)^19,21^. The expression of GSDM family members are highly cell type and tissue-specific, so they have differentiation-status specific roles^21,22^. GSDM family are a class of pore-forming proteins^23^. Previous studies have shown that GSDMA, GSDMB, GSDMD, GSDME, and GSDMF perform a crucial function in cell death, inflammation, and autoimmunity^19,24^. But the biological function of GSDMC has not been identified^25^.

GSDMC is also known as leucine zipper-containing extranuclear factor^26,27^. Several investigations show that ultraviolet (UV) radiation increased the expression of GSDMC, and GSDMC may have an instrumental function in the triggering of ERK and JNK pathways which result in UV-induced MMP-1 expression^28,29^. Some scholars suggest the expression levels of GSDMC may be associated with the development of lumbar spinal stenosis. Past research reports have indicated that GSDMC could have an important function during tumorigenesis including cell proliferation in colorectal cancer as well as enhanced metastatic prospects in melanoma cells^28,30^. Recent studies demonstrate that the transcription of GSDMC is enhanced by PD-L1 interacts with p-Stat3 as well as its nuclear translocation under hypoxia^12^. The metabolite α-KG induces death receptor 6-activated caspase-8 which activates the GSDMC-dependent pyroptosis pathway in cancer cells, causing tumor necrosis^15^. Nevertheless, the exact functions of GSDMC are still inadequately investigated and need to be further elucidated.

**Figure 1:**
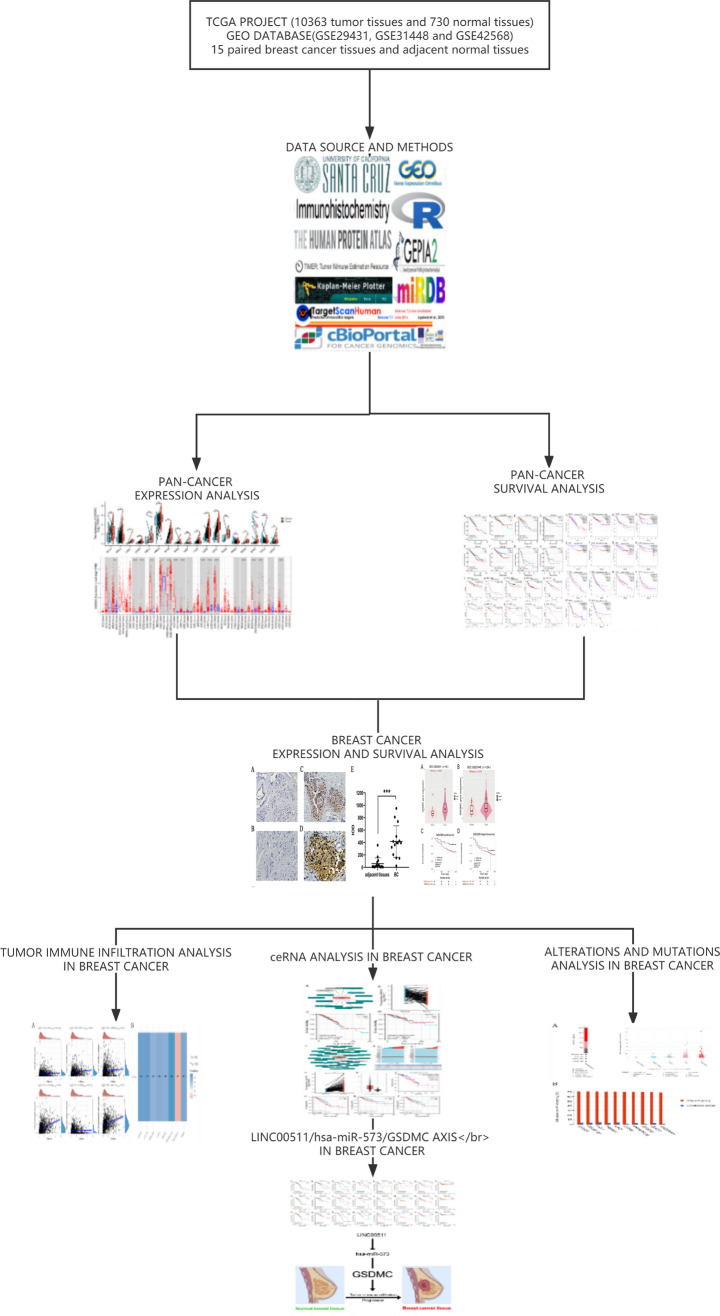
Analysis workflow and results of this research.

In this research, we systematically examined the GSDMC expression and its link to the prognosis of pan-tumors utilizing multidimensional data from the TCGA and GEO databases. Validation immunohistochemical experiments were performed. Next, we identified microRNAs (miRNAs) and long noncoding RNAs (lncRNAs) that accounts for GSDMC in BRCA. Furthermore, we demonstrated the associations between GSDMC expression and immune infiltrates, immune checkpoints, and immune markers in BRCA. Finally, we employed the cBioPortal online tool to evaluate modifications, mutations, and pathways of GSDMC in BRCA. This research illustrated that ncRNAs-mediated upregulation of GSDMC was linked to dismal prognosis and also exhibited a correlation with tumor immune cell infiltration in BRCA. Figure 1 exhibited the overall design, workflow and results of this study.

## Results

### Evaluation of GSDMC expression in different cancers and non-cancer normal tissues

**Figure 2:**
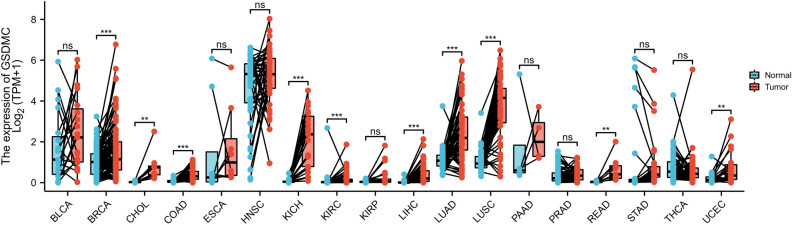
The transcription levels of GSDMC in different cancers from TGGA database. *P < 0.05, **P < 0.01, ***P < 0.001.

**Figure 3:**
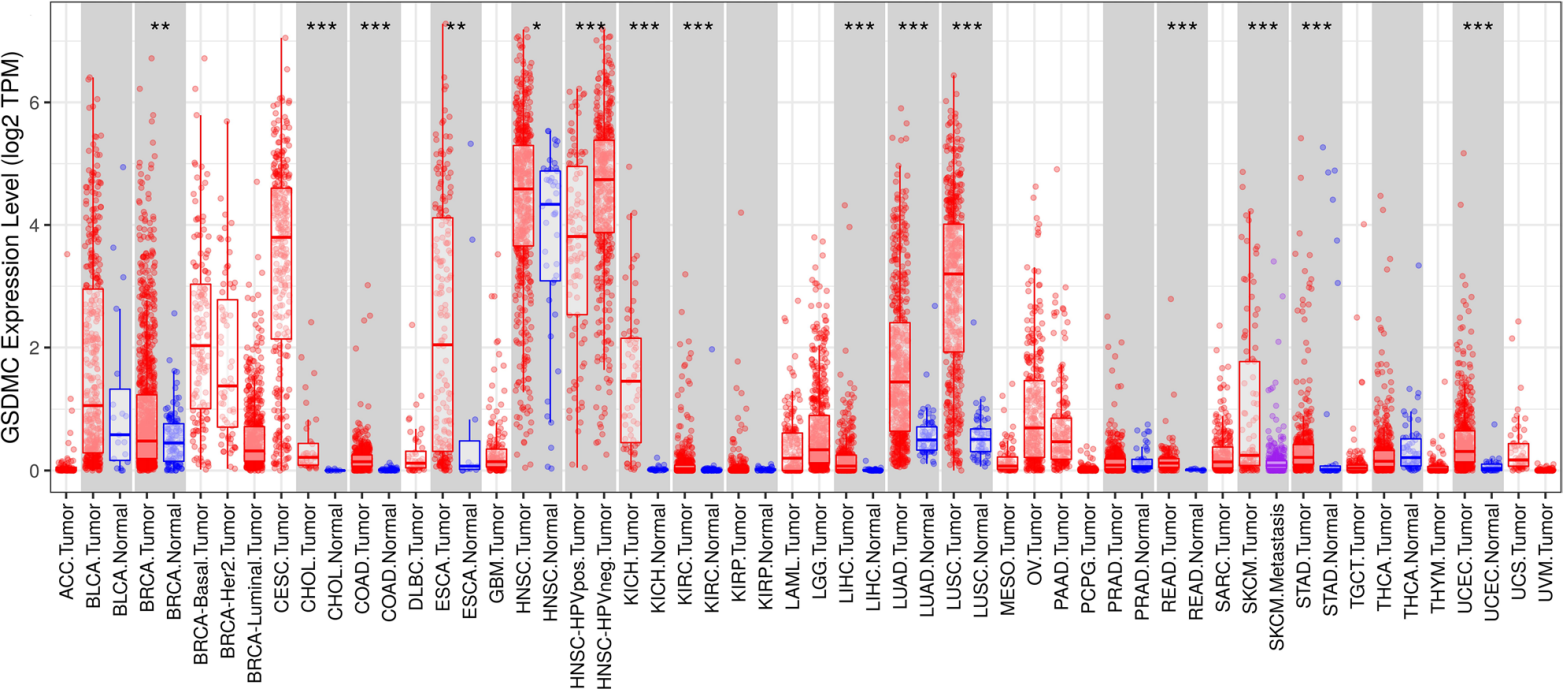
GSDMC expression levels in different tumor types from TCGA database were determined by TIMER. *P < 0.05, **P < 0.01, ***P < 0.001.

First, via the TCGA dataset, we examined the levels of mRNA expression of GSDMC in 10,363 tumor tissues and 730 adjoining tissues from 18 kinds of cancer. The outcomes illustrated that the levels of GSDMC expression were higher than normal tissues control in BRCA, Cholangio carcinoma (CHOL), colon adenocarcinoma (COAD), Lung squamous cell carcinoma (LUSC), Kidney Chromophobe (KICH), Uterine Corpus Endometrial Carcinoma (UCEC), Rectum adenocarcinoma (READ), Kidney renal clear cell carcinoma (KIRC), Lung squamous cell carcinoma (LUSC), Liver hepatocellular carcinoma (LIHC), and Lung adenocarcinoma (LUAD) (Fig. 2), however, no significant difference of GSDMC in Bladder Cancer (BLCA), stomach adenocarcinoma (STAD), kidney renal papillary cell carcinoma (KIRP), Esophageal carcinoma (ESCA), Pancreatic adenocarcinoma (PAAD), Head and Neck squamous cell carcinoma (HNSC), and thyroid Cancer (THCA) were observed (Fig. 2). In order to validate these, we further assessed how GSDMC expression differs in pan tumor types in TCGA databases via Tumor Immune Estimation Resource (TIMER). In TIMER databases, we found that GSDMC expression was significantly higher in UCEC, BRCA, STAD, CHOL, READ, COAD, ESCA, LUSC, HNSC, LUAD, KICH, LIHC, and KIRC as opposed to adjoining non-cancer normal tissues (Fig. 3). However, GSDMC expression was no significant difference in BLCA, KIRP, prostate adenocarcinoma (PRAD), and THCA as opposed to adjoining non-cancer normal tissues (Fig. 3). Taken together, the upregulation of GSDMC was found in UCEC, BRCA, READ, CHOL, LUSC, COAD, LUAD, KICH, LIHC, and KIRC. These data exhibited differential expressions of GSDMC in these types of cancer.

### The correlation between GSDMC expression and cancer patient prognosis

**Figure 4:**
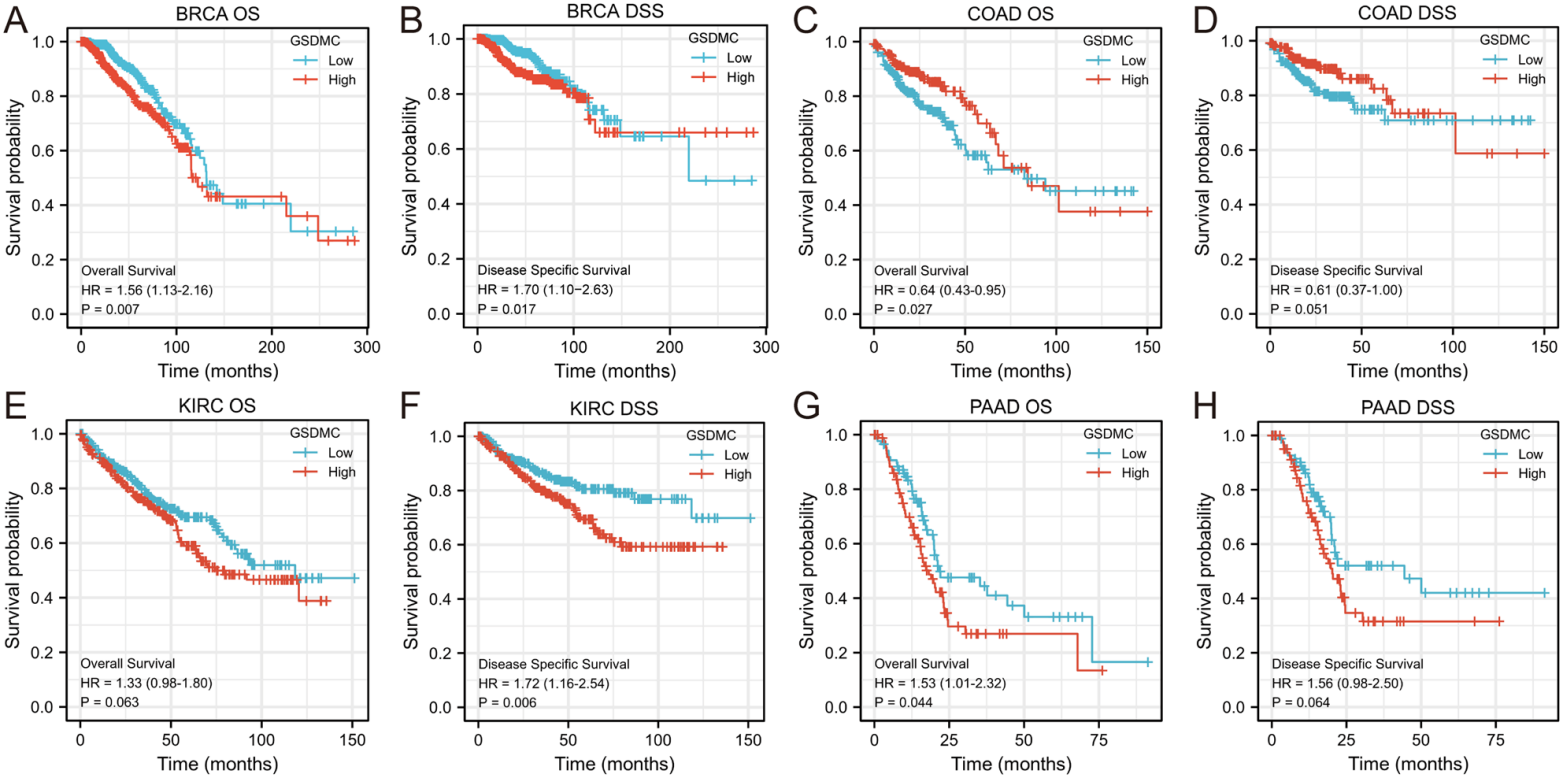
Prognostic analysis of GSDMC mRNA expression levels in different tumor types in the TCGA database (**A–H**). *P < 0.05, **P < 0.01, ***P < 0.001. *OS* overall survival, *DSS* disease-specific survival.

We utilized the KM survival analysis with the log-rank test to assess the correlation between the levels of GSDMC expression and the survival (overall survival (OS) and disease-specific survival (DSS)) of patients in pan-tumors and normal tissue types in the TGGA database (Fig. S1). We found that BRCA, COAD, and PAAD exhibited a considerable relationship between the levels of GSDMC expression and patients' OS, and the levels of GSDMC expression were linked to patients' DSS in BRCA and KIRC (Fig. 4). Higher GSDMC expression levels have significance relationship with poorer prognosis in BRCA (OS, HR 1.56 (1.13–2.16), *P* = 0.007; DDS, HR 1.70 (1.10–2.63), *P* = 0.017) (Fig. 4A,B). Elevated levels of mRNA expression in GSDMC were linked to poorer prognosis PAAD (OS, HR 1.53 (1.01–2.32), *P* = 0.044), but showed no significant correlations with DSS in PAAD (DSS, HR 1.56 (0.98–2.50), *P* = 0.064) (Fig. 4C,D). In KIRC, high GSDMC expressions were associated with poorer DSS (DSS, HR 1.72 (1.16–2.54), *P* = 0.006), but no significant associations with OS (OS, HR 1.33 (0.98–1.80), *P* = 0.063) (Fig. 4E,F). On the contrary, we discovered that elevated expression levels of GSDMC were linked to improved OS in COAD (OS, HR 0.64 (0.43–0.95), *P* = 0.027), but showed no significant relationships with DSS in COAD (DSS, HR 0.61 (0.37–1.00), P = 0.007) (Fig. 4G,H). No substantial correlations were observed between GSDMC expression levels and patient prognosis in other types of cancers (Fig. S1).

**Figure 5:**
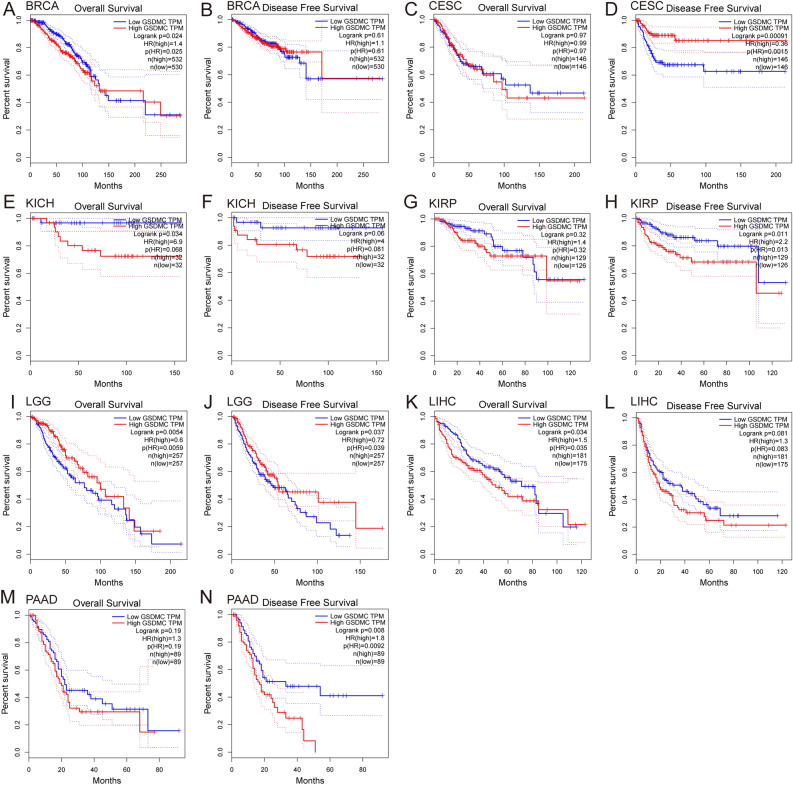
Prognostic analysis of GSDMC mRNA expression levels in various human cancers determined by GEPIA database (**A–N**). *OS* overall survival, *DFS* disease-free survival.

**Figure 6:**
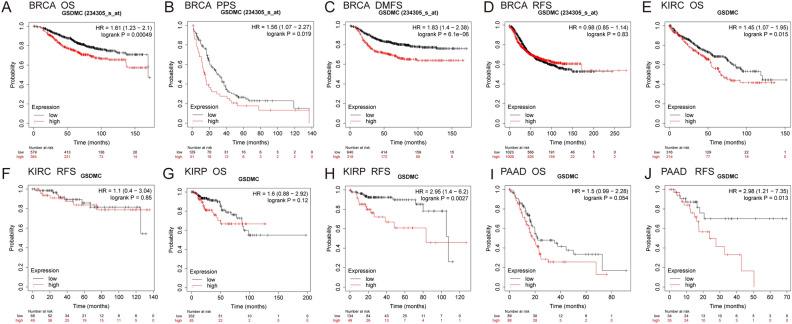
Kaplan–Meier survival curves comparing the high and low expression of GSDMC in different types of cancers in the Kaplan–Meier plotter databases (**A–J**). *OS* overall survival, *DFS* disease-free survival, *RFS* relapse-free survival, *PPS* post-progression survival, *DMFS* distant metastasis-free survival.

Next, we validated the link between the levels of GSDMC expression and patient prognosis in multiple cancer kinds via the GEPIA database and KM plotter database (Figs. 5, 6, Figs. S2, S3). In GEPIA, we discovered that elevated mRNA expression levels of GSDMC were linked to worse OS in BRCA (OS, HR = 1.4, *P* = 0.024), KICH (OS, HR 6.9, *P* = 0.034), LIHC (OS, HR 1.5, *P* = 0.034) and with poorer DFS in KIRP (DFS, HR 2.2, *P* = 0.011), and PAAD (DFS, HR 1.8, *P* = 0.008) (Fig. 5A,E,H,K,N). On the contrary, elevated mRNA expression levels of GSDMC were linked to improved prognosis in LGG (OS HR 0.6, *P* = 0.0054; DFS, HR = 0.72, *P* = 0.037) and better DFS (DFS HR 0.36, *P* = 0.00091) in CESC (Fig. 5D,I,J), but showed no significant correlation in other tumors (Fig. 5B,C,F,G,L,M, Fig. S2). Then we utilized the KM plotter to explore the link between the levels of GSDMC expression and patient prognosis in various pan-cancer types. As depicted in Fig. 6, consistency with the results of above, higher GSDMC expression levels had substantial link to worse prognosis in BRCA (OS, HR 1.61 (1.23–2.1), *P* = 0.00049; distant metastasis-free survival (DMFS), HR 1.83 (1.4–2.38), *P* = 0.000061; post-progression survival (PPS), HR 1.56 (1.07–2.27), *P* = 0.019) (Fig. 6A–C). In PAAD and KIRP, high GSDMC expressions were associated with poorer relapse-free survival (RFS) (PAAD, HR = 2.98 (1.21–7.35), *P* = 0.013; KIRP, HR 2.95 (1.4–6.2), *P* = 0.0027), but no significant associations with OS (PAAD, HR 1.5 (0.99–2.28), *P* = 0.054; KIRP, HR 1.6 (0.88–2.92), *P* = 0.12) (Fig. 6G–J). Meanwhile, elevated levels of GSDMC expression were linked to improved OS in KIRC (OS, HR 1.45 (1.07–1.95), *P* = 0.015) (Fig. 6E), but showed no significant relationships with RFS in COAD (RFS, HR 1.1 (0.4–3.04), *P* = 0.85) (Fig. S3).

Taken together, the combination of OS, RFS, DSS, DMFS, and PPS and concern of bias, our findings illustrated the expression levels and prognostic value of GSDMC in several kinds of cancers, GSDMC might perform as a negative prognostic biomarker in BRCA patients. However, much further research is needed to investigate the link between the expression of GSDMC and cancer patient prognosis in other kinds of cancers, including PAAD, COAD, KICH, etc.

### Protein expression analysis and prognosis analysis of GSDMC in BC

**Figure 7:**
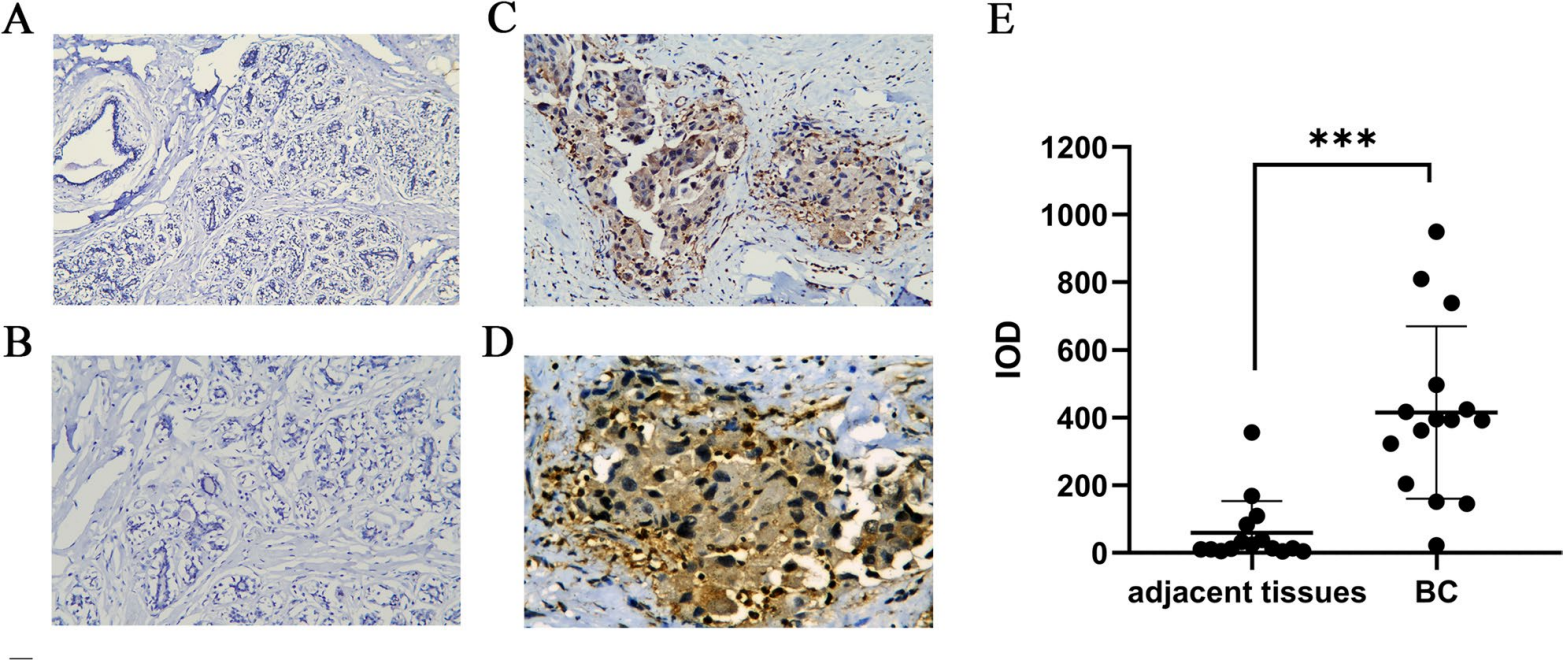
GSDMC protein expression analysis. (**A**) GSDMC protein expression in normal breast tissue (× 200 magnification); (**B**) GSDMC protein expression in normal breast tissue (× 400 magnification); (**C**) GSDMC protein expression in BC tumor tissue (× 200 magnification); (**D**) GSDMC protein expression in BC tumor tissue (× 400 magnification); (**E**) quantification of immunostains for GSDMC by IOD analysis. ***P < 0.001. *BC* breast cancer.

Subsequently, IHC was performed to validate the expression of GSDMC in 15 pairs of BC tumor tissues and corresponding adjacent normal tissues. IHC staining analysis exhibited that GSDMC was mainly localized in the cytoplasm of cancer cells, and brown staining indicated positive staining (Fig. 7C,D). Weak to no expression of GSDMC were observed in the normal tissues (Fig. 7A,B). Statistical analysis revealed that GSDMC was also expressed significantly highly in BC tissues than in the adjacent non-tumor tissues (*P* < 0.001) (Fig. 7E). Survival analysis showed that high protein expression of GSDMC had worse PFS in BC, however, there was no statistically significant (*P* > 0.05) (Fig. S4A).

### GSDMC expression and prognosis analysis of BC in GEO database

**Figure 8:**
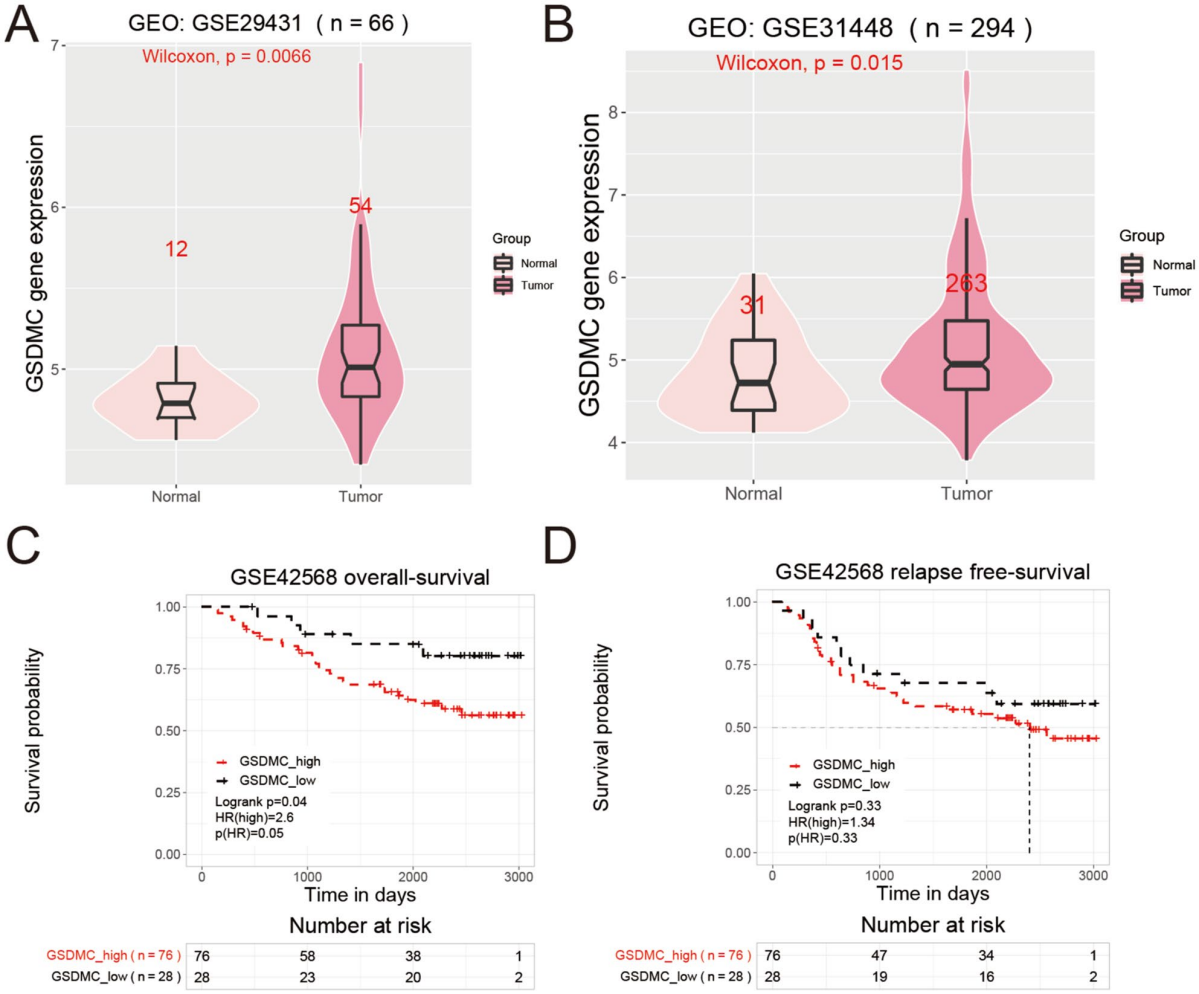
(**A,B**) Relative mRNA expression of GSDMC in BC and paired normal tissues from GEO database. ((**A**) in GSE31192; (**B**) in GSE42568). (**C,D**) Kaplan–Meier survival curves comparing the high and low expression of GSDMC in BC from GEO database (in GSE31192).

Then, we utilized GEO database to perform expression and survival analyses of GSDMC in BC. Expression analysis indicated that mRNA expression levels of GSDMC were significantly higher in BC tissues than in normal control tissues in GSE29431 and GSE31448 (*P* < 0.05) (Fig. 8A,B). Survival analysis of GSE42568 exhibited significant relationships between high expressions of GSDMC and worse OS in BC patients (*P* < 0.05) (Fig. 8C). Whereas high mRNA expressions of GSDMC showed no significant relationships with RFS in BC (*P* > 0.05) (Fig. 8D). Thus, further experimental validation is needed.

### Prediction and analysis of potential miRNA candidates of GSDMC

**Figure 9:**
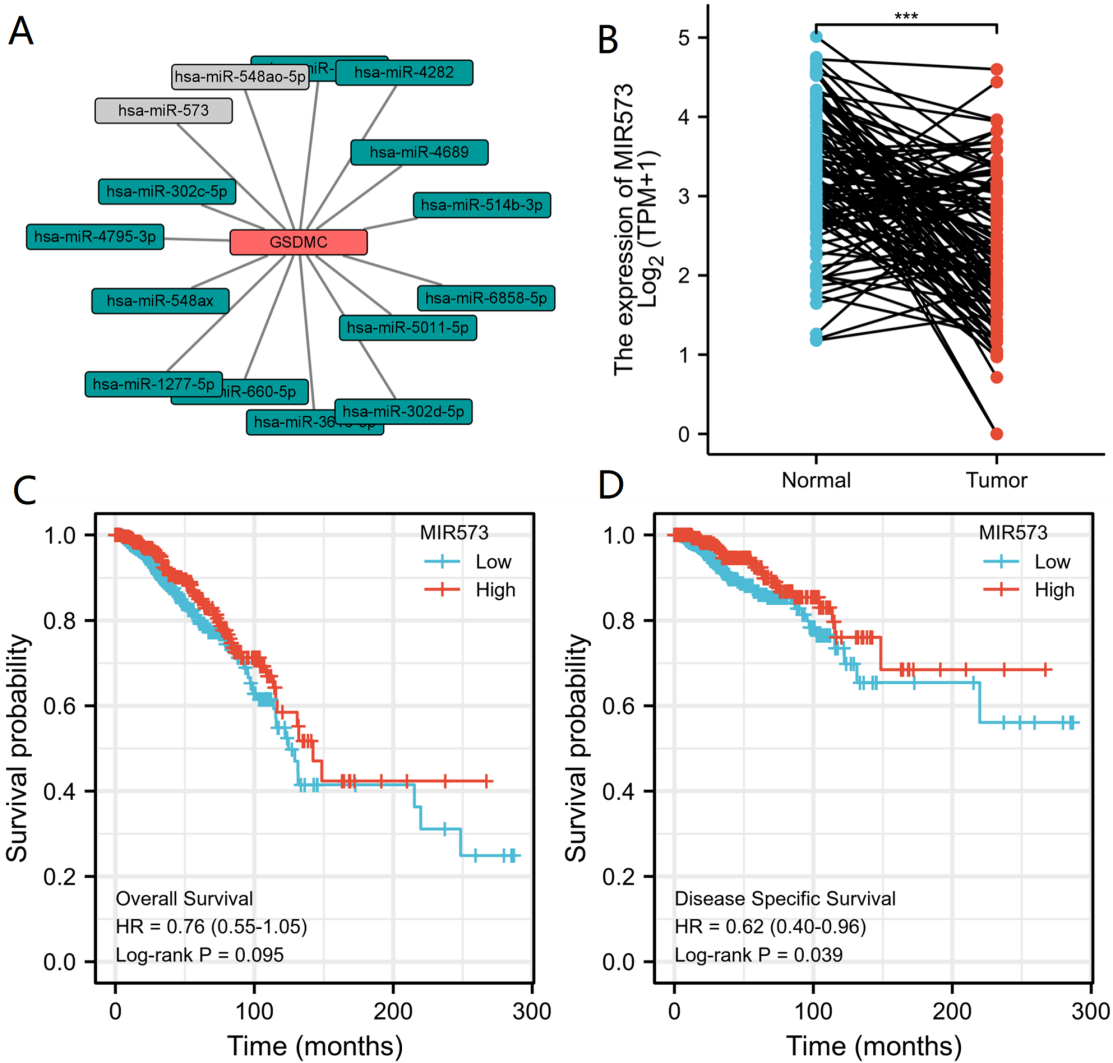
(**A**) miRNA-GSDMC regulatory network; (**B**) hsa-miR-573 differentially expression levels in normal breast tissues and breast cancer tissues in TCGA database; (**C**) the overall survival (OS) analysis for hsa-miR-573 in BRCA in TCGA database; (**D**) the disease-specific survival (DSS) analysis for hsa-miR-573 in BRCA in TCGA database. *P < 0.05, **P < 0.01, ***P < 0.001. *OS* overall survival, *DSS* disease-specific survival.

ncRNAs are well-recognized for regulating gene expression at almost every step^31–33^. In order to determine whether GSDMC was regulated by various ncRNAs in BRCA, we forecasted potential candidate miRNAs that might bind to GSDMC and ultimately identified 15 miRNAs. We used various target gene forecasting website, comprising of miRDB, miRmap, TargetScan, miRcode, miRWalk and DIANA-microT to establish a miRNA-GSDMC regulatory network (Fig. 9A). Because the action mechanism of miRNAs negatively modulates the GSDMC expression at the post-transcriptional level, there should be a negative relationship between GSDMC and miRNA in BRCA. So, we investigated the relationship between GSDMC and 15 miRNAs in BRCA via the TCGA database. As a result, we found that GSDMC was significantly negatively associated with hsa-miR-573 (MIR573) and positively linked to hsa-miR-548ao-5p (MIR548AO) in BRCA (*P* < 0.001, Fig. 9A, Table 1). Meanwhile, no statistical expression relations were observed between GSDMC and other miRNAs (Fig. 9A, Table 1). Then we explored the miRNA expression levels of hsa-miR-573 in 1109 tumor tissues and 113 adjoining tissues from BRCA in the TGGA dataset. The outcomes indicated that the levels of hsa-miR-573 expression were lower than normal tissue control in BRCA (Fig. 9B). Subsequently, we ascertained the link between hsa-miR-573 expression levels and BRCA patient prognosis in the TCGA database. We discovered that elevated miRNA expression level of hsa-miR-573 was considerably linked to improved DSS in BRCA (DSS, HR 0.62 (0.40–0.92), *P* = 0.039) (Fig. 9D). Meanwhile, the hsa-miR-573 expression level was linked to improved OS in BRCA, but it was not statistically significant (OS, HR 0.76 (0.55–1.05), *P* = 0.095) (Fig. 9C). Taking together survival analysis, expression analysis, and correlation analysis, we suggested that hsa-miR-573 might serve as potential regulating miRNA for GSDMC in BRCA.

**Table 1:** The relationship between of GSDMC expression and potential candidate miRNAs expression in BRCA.

GENE	miRNA	R-value (Pearson)	*P*-value (Pearson)	*P*.adjust	R-value (Spearman)	*P*-value (Spearman)	*P*.adjust
GSDMC	hsa-miR-4795-3p	− 0.050	0.096	0.416	− 0.070	0.020*	0.26
GSDMC	hsa-miR-548ax	0.026	0.394	0.7046	0.022	0.461	0.72655
GSDMC	hsa-miR-548ao-5p	0.214	< 0.001***	< 0.001***	0.208	< 0.001***	< 0.001***
GSDMC	hsa-miR-4689	0.010	0.744	0.806	0.021	0.475	0.7265
GSDMC	hsa-miR-6858-5p	0.019	0.525	0.7046	0.016	0.606	0.7457
GSDMC	hsa-miR-302d-5p	− 0.013	0.661	0.7811	− 0.034	0.260	0.7265
GSDMC	hsa-miR-4282	0.022	0.467	0.7046	− 0.023	0.440	0.7265
GSDMC	hsa-miR-5011-5p	0.066	0.027	0.351	0.029	0.343	0.7265
GSDMC	hsa-miR-302c-5p	− 0.020	0.508	0.7046	− 0.029	0.330	0.7265
GSDMC	hsa-miR-5702	0.004	0.886	0.886	0.003	0.911	0.911
GSDMC	hsa-miR-1277-5p	0.034	0.261	0.689	0.020	0.503	0.7265
GSDMC	hsa-miR-660-5p	− 0.033	0.265	0.689	− 0.014	0.631	0.7457
GSDMC	hsa-miR-573	− 0.209	< 0.001***	< 0.001***	− 0.203	< 0.001***	< 0.001***
GSDMC	hsa-miR-3616-5p	0.018	0.542	0.7046	0.042	0.167	0.7265
GSDMC	hsa-miR-514b-3p	0.051	0.090	0.416	0.011	0.705	0.7637

*P < 0.05 (5e-02); **P < 0.01 (1e−02); ***P < 0.001 (1e−03).

### Evaluation of potential candidate lncRNAs of hsa-miR-573

**Figure 10:**
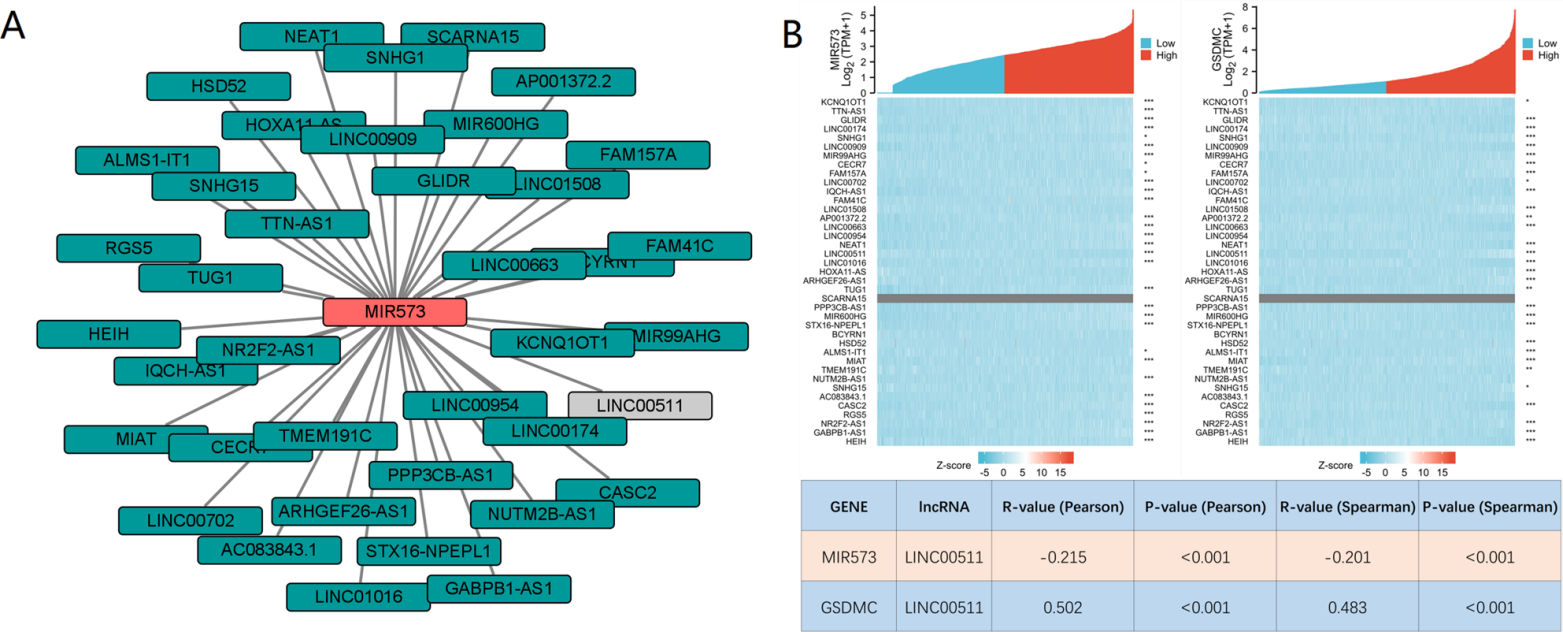
(**A**) lncRNA-hsa-miR-573 regulatory network; (**B**) correlation analysis of hsa-miR-573 expression and 39 lncRNAs in BRCA in TCGA database. *P < 0.05, **P < 0.01, ***P < 0.001.

**Figure 11:**
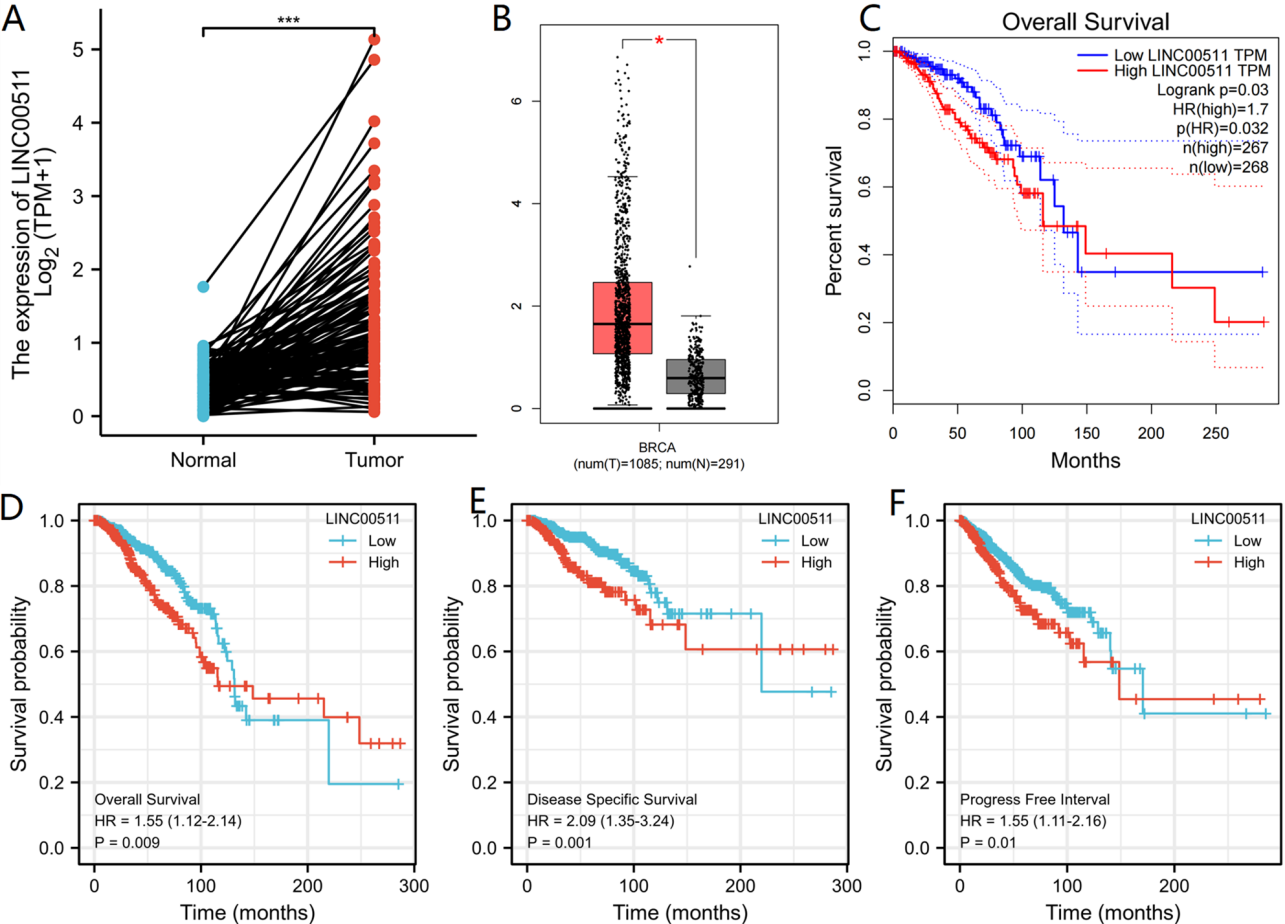
(**A**) LINC00511 differentially expression levels in normal breast tissues and breast cancer tissues in TCGA database; (**B**) LINC00511 differentially expression levels in normal breast tissues and breast cancer tissues determined by GEPIA database; (**C**) the overall survival (OS) analysis for hsa-miR-573 in BRCA determined by GEPIA database in TCGA database; (**D–F**) overall survival (OS), disease-specific survival (DSS) and progress free interval (PFI) analysis for LINC00511 in BRCA in TCGA database. *P < 0.05, **P < 0.01, ***P < 0.001.

Then we forecasted upstream potential lncRNAs that interact with hsa-miR-573 by using DIANA-LncBase v.2. A total of 39 possible lncRNAs were selected as candidate lncRNAs in breast tissues and mammary gland tissues (Threshold > 7). A lncRNA-hsa-miR-573 regulatory network was visualized using Cystoscope software (Fig. 10A). The competitive endogenous RNA (ceRNA) hypothesis suggests that lncRNA reduces the suppressive miRNA-effect on target-mRNAs. Therefore, in the ceRNA network, lncRNA should be positively correlated with target mRNA while lncRNA should be negatively correlated with target miRNA. Correlation analysis of hsa-miR-573 expression and 39 lncRNAs was done in the TGGA breast cancer database. The results highlighted that only LINC00511 was negatively associated with hsa-miR-573, and positively associated with GSDMC (Fig. 10B). Then we took expression analysis in the TCGA set. As a result, we discovered that the levels of LINC00511 expression were considerably upregulated in BRCA as opposed to normal controls (Fig. 11A). We used the GEPIA database to validate, GEPIA results were consistent with the aforementioned results (Fig. 11B). Subsequently, we assessed the prognostic values of LINC00511 in BRCA. We observed that elevated mRNA expression levels of hsa-miR-573 were considerably linked to poorer OS (HR 1.55 (1.12–2.14), *P* = 0.009), DSS (HR 2.09 (1.35–3.24), *P* = 0.001) and (Progress free interval) PFI (HR 1.55 (1.11–2.16), *P* = 0.01) in BRCA (Fig. 11D–F). In GEPIA database, elevated LINC00511 expression was also substantially linked to worse OS (HR 1.7, *P* = 0.03) in BRCA (Fig. 11C). External validation was carried out using two GEO databases (GSE29431, GSE42568). In GSE29431, the levels of LINC00511 expression were considerably upregulated in BC as opposed to normal controls (*P* = 0.00076) (Fig. S4B). The survival analysis showed high expression of LINC00511 might increase the risk of death for BC; however, this was not statistically significant (*P* = 0.064) (Fig. S4C). Taking into account survival analysis, expression analysis, as well as correlation analysis, LINC00511 might be the key potential upstream lncRNA of the GSDMC/hsa-miR-573 axis in BRCA.

### Association of GSDMC, hsa-miR-573 and LINC00511 expression levels and prognosis in patients with molecular subtyping of BRCA

**Figure 12:**
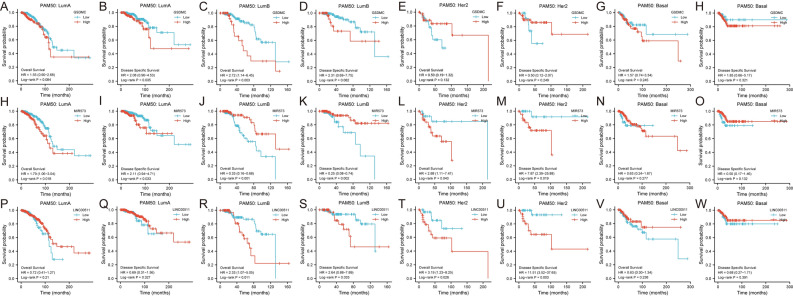
Association of GSDMC, hsa-miR-573 and LINC00511 expression levels and prognosis in patients with molecular subtyping of BRCA (**A–W**). *LumA* luminal A, *LumB* luminal B, *N* normal-like, *Her2* Her-2 enrichment, *Basal* basal-like breast cancer.

Molecular subtyping provides precision treatment guidance in BRCA. Thus, we utilized KM plotter to assess the association between expression levels of GSDMC, hsa-miR-573, LINC00511 and prognosis in patients with differently molecular subtyping of BRCA (Fig. 12). Interestingly, results showed that only in luminal B BRCA, high mRNA expression levels of GSDMC and LINC00511 were significantly associated with dismal prognosis (*P* < 0.05), as well as high mRNA expression levels of hsa-miR-573 were significantly correlated with good prognosis (*P* < 0.05) (Fig. 12C,J,K,R,S). In addition, high expression of GSDMC in luminal A BRCA exhibited a significant correlation with worse DSS rates, and elevated expression of LINC00511 exhibited a significant relationship with higher OS and DSS rate in HER2 enriched BRCA (*P* < 0.05) (Fig. 12B,T,U). Notably, high mRNA expression levels of hsa-miR-573 exhibited significant correlation with increased OS and DSS rates in luminal A and HER2 enriched BRCA patients (*P* < 0.05) (Fig. 12H,I,L,M). This intriguing phenomenon requires additional experimental confirmation. No substantial correlations were observed in other types (Fig. 12A,E–H,N,O–Q,V,W).

### The expression of GSDMC associated with immune cell infiltration in BRCA

**Figure 13:**
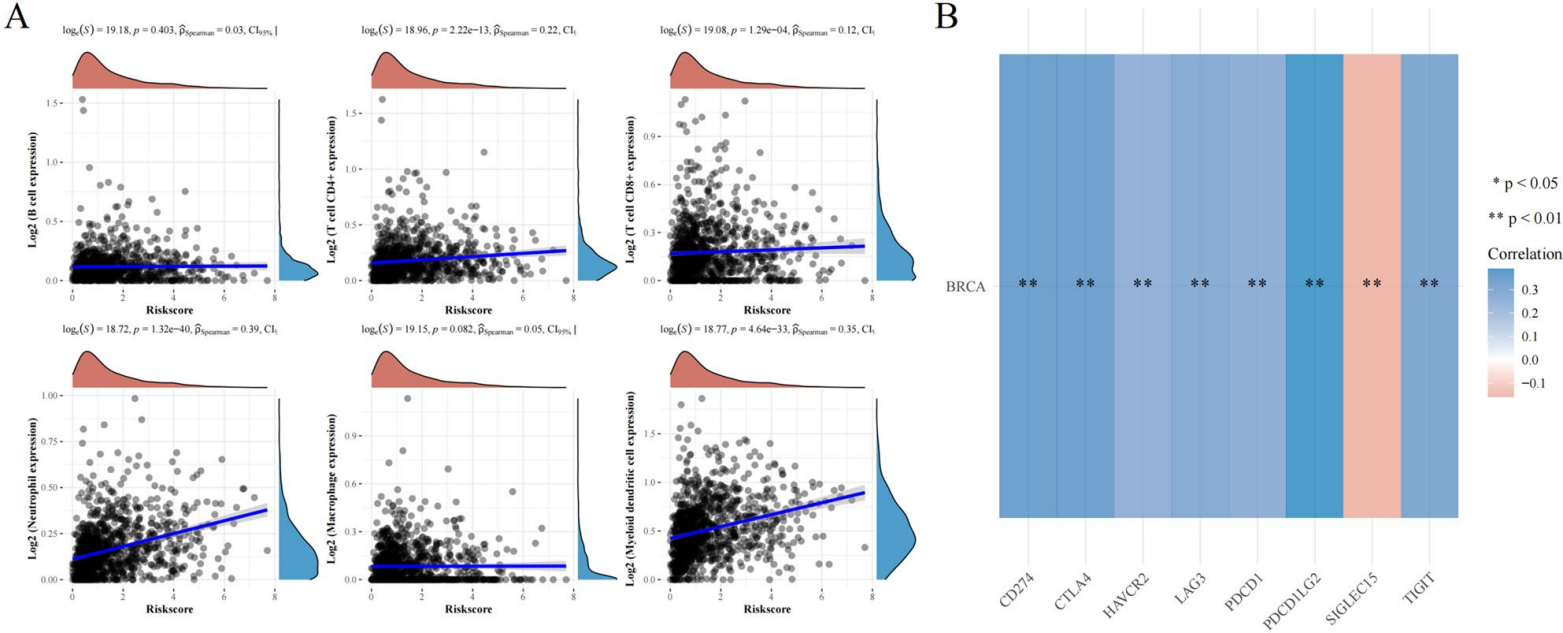
(**A**) The relationship between GSDMC expression levels and immune cell infiltration in BRCA via TCGA database; (**B**) the relationship between GSDMC expression levels and immune checkpoints in BRCA via TCGA database. *P < 0.05, **P < 0.01, ***P < 0.001.

GSDMC, which belongs to the Gasdermin superfamily, is believed to participate in the modulation of epithelial cell immune-related functions^30^. Therefore, we examined the link between the levels of GSDMC expression and immune cell infiltration in BRCA. The observed finding suggested that GSDMC expression were considerably correlated with the level of CD4+ T cells (R = 0.22, *P* = 2.22e−13), CD8 + T cells level (R = 0.12, *P* = 1.29e−04), level of neutrophils (R = 0.39, *P* = 1.32e−40), level of myeloid dendritic cells (R = 0.35, *P* = 4.64e−33), whereas no association was observed with the levels of B cells and macrophages in BRCA (Fig. 13A). These results illustrated that GSDMC performed a vital function in the regulation of immune cell infiltration in BRCA. In BRCA, GSDMC had a specifically solid function in tumor purity and the infiltration of myeloid dendritic cells, neutrophils. CD4+ T cells, and CD8+ T cells.

### The link between GSDMC and immune markers expression in BRCA

Considering that GSDMC performs a crucial function in the modulation of immune cell infiltration in BRCA, we explored the link between the expression of GSDMC and various immunological markers subsets such as M1 and M2 macrophages, exhausted T cells, total B cells, T cells, TAMs, monocytes, CD8+ T cells, natural killer cells (NK cells), neutrophils, myeloid dendritic cells (MDCs), tfh cells, Tregs, Th17 cells Th1 cells, and Th2 cells by the TIMER databases, Tumor purity were adjusted. Our finding showed significant positive correlations between GSDMC expression and B cells (CD19, CD79A), T cells (CD2, CD3D, CD3E), CD8+ T cell (CD8A, CD8B), Treg markers (CCR8, FOXP3), monocyte markers (CD86, CD115), Th17 markers (IL17A, STAT3), TAM markers (IL10, CD68, CCL2), Th2 markers (IL13, STAT5A) M1 macrophage markers (COX2, IRF5), M2 macrophage markers (MS4A4A, VSIG4, CD163), NK cell markers (KIR2DL1, KIR2DL3, KIR2DL4, KIR3DL1, KIR3DL2, KIR3DL3, KIR2DL4), neutrophils markers (CCR7, CD11b), DC markers (CD11C, HLA-DQB1, HLA-DRA, HLA-DPA1, BDCA-4, HLA-DPB1), Th1 markers (TNF-α, STAT1, IFN-γ, STAT4, T-bet), Tfh markers (IL21), T cell exhaustion markers (GZMB, TIM-3, LAG3, CTLA4, PD-1) in BRCA (*P* < 0.01, Table 2). Remarkably, in BRCA, the levels of GSDMC expression were considerably negatively linked to Th2 markers (GATA3, STAT6) (*P* < 0.01, Table 2). These findings supported that GSDMC was considerably related to immune infiltrating cells as well as the immune microenvironment in BRCA.

**Table 2:** Correlation analysis between GSDMC and relate genes and markers of immune cells in TIMER.

Description	Gene markers	BRCA					
		None	*P*	*P*.adjust	Purity	*P*	*P*.adjust
		Cor			Cor		
CD8 + T cell	CD8A	0.168	2.05e−08***	2.73E−08	0.098	2.06e−03*	0.002507826
	CD8B	0.218	2.81e−13	7.15E−13	0.157	6.55e−07***	1.53E−06
T cell (general)	CD3D	0.225	4.60e−14***	1.29E−13	0.15	2.05e−06***	3.59E−06
	CD3E	0.213	9.85e−13***	2.12E−12	0.136	1.73e−05***	2.85E−05
	CD2	0.24	7.77e−16***	3.11E−15	0.171	5.58e−08***	1.56E−07
B cell	CD19	0.2	2.24e−11***	3.69E−11	0.118	1.85e−04**	0.000259
	CD79A	0.194	8.35e−11***	1.30E−10	0.116	2.49e−04**	0.000332
Monocyte	CD86	0.29	9.83e−23***	9.17E−22	0.247	2.48e−15***	2.31E−14
	CD115 (CSF1R)	0.154	2.68e−07***	3.13E−07	0.085	7.38e−03*	0.007653333
TAM	CCL2	0.347	1.92e−32***	5.38E−31	0.229	5.30e−22***	1.48E−20
	CD68	0.256	6.32e−18***	2.95E−17	0.216	6.15e−12***	2.87E−11
	IL10	0.265	3.91e−19***	2.74E−18	0.221	1.69e−12***	9.46E−12
M1 Macrophage	INOS (NOS2)	0.071	1.83e−02	0.0183	0.065	4.06e−02	0.0406
	IRF5	0.145	1.33e−06***	1.49E−06	0.109	5.76e−04**	0.000733091
	COX2 (PTGS2)	0.238	1.27e−15***	4.45E−15	0.182	7.92e−09***	3.17E−08
M2 Macrophage	CD163	0.263	6.83e−19***	3.82E−18	0.224	8.70e−13***	6.09E−12
	VSIG4	0.168	2.15e−08***	2.74E−08	0.121	1.23e−04**	0.000181263
	MS4A4A	0.205	6.16e−12***	1.08E−11	0.155	9.27e−07***	1.83E−06
Neutrophils	CD66b (CEACAM8)	0.078	9.91e−03*	0.0102	0.087	6.10e−03*	0.006569231
	CD11b (ITGAM)	0.21	2.03e−12***	3.79E−12	0.156	7.43e−07***	1.60E−06
	CCR7	0.178	2.50e−09***	3.50E−09	0.095	2.80e−03*	0.003266667
Naturalkiller cell	KIR2DL1	0.18	1.84e−09***	2.71E−09	0.155	9.81e−07***	1.83E−06
	KIR2DL3	0.217	3.34e−13***	7.79E−13	0.177	1.98e−08***	6.16E−08
	KIR2DL4	0.321	1.06e−27***	1.48E−26	0.279	3.39e−19***	4.75E−18
	KIR3DL1	0.212	1.12e−12***	2.24E−12	0.163	2.38e−07***	6.06E−07
	KIR3DL2	0.232	6.20e−15***	1.93E−14	0.177	1.87e−08***	6.16E−08
	KIR3DL3	0.109	2.96e−04**	0.0003	0.09	4.74e−03*	0.0053088
	KIR2DS4	0.167	2.58e−08***	3.14E−08	0.133	2.69e−05***	4.18E−05
Dendritic cell	HLA-DPB1	0.141	2.83e−06***	3.73E−06	0.047	1.37e−01	0.137
	HLA-DQB1	0.185	6.81e−10***	1.04E−09	0.121	1.27e−04**	0.000167409
	HLA-DRA	0.24	7.54e−16***	1.68E−15	0.17	7.09e−08***	1.37E−07
	HLA-DPA1	0.157	1.68e−07***	2.44E−07	0.075	1.75e−02	0.019519231
	BCDA-1 (CD1C)	0.042	1.63e−01	0.163	− 0.084	8.02e−03*	0.0093032
	BDCA-4 (NRP1)	0.126	2.87e−05***	3.40E−05	0.086	6.65e−03*	0.008035417
	CD11c (ITGAX)	0.247	8.33e−17***	2.20E−16	0.19	1.59e−09***	3.84E−09
Th1	T-bet (TBX21)	0.232	7.09e−15***	1.47E−14	0.163	2.24e−07***	3.82E−07
	STAT4	0.227	2.37e−14***	4.58E−14	0.17	7.58e−08***	1.37E−07
	STAT1	0.248	7.94e−17***	2.20E−16	0.225	6.55e−13***	1.90E−12
	IFN-γ (IFNG)	0.292	4.57e−23***	1.89E−22	0.245	4.22e−15***	1.68E−14
	TNF-α (TNF)	0.281	2.10e−21***	7.61E−21	0.245	4.64e−15***	1.68E−14
Th2	GATA3	0.498	4.89e−70***	1.42E−68	− 0.472	2.37e−56***	6.87E−55
	STAT6	0.201	1.54e−11***	2.63E−11	− 0.237	4.13e−14***	1.33E−13
	STAT5A	0.126	2.93e−05***	3.40E−05	0.068	3.17e−02	0.034048148
	IL13	0.191	1.61e−10***	2.59E−10	0.157	6.78e−07***	1.09E−06
Tfh	BCL6	0.081	7.06e−03*	0.007582963	0.054	9.08e−02	0.094042857
	IL21	0.213	1.02e−12***	1.85E−12	0.179	1.34e−08***	2.78E−08
Th17	STAT3	0.127	2.24e−05***	2.82E−05	0.112	3.95e−04**	0.000498043
	IL17A	0.15	5.54e−07***	7.65E−07	0.129	4.46e−05***	6.81E−05
Treg	FOXP3	0.311	4.90e−26***	3.55E−25	0.254	4.14e−16***	2.00E−15
	CCR8	0.299	3.56e−24***	2.06E−23	0.263	3.71e−17***	2.69E−16
	STAT5B	0.095	1.55e−03*	0.001728846	− 0.123	1.07e−04**	0.000147762
	TGFβ (TGFB1)	0.046	1.30e−01	0.134642857	− 0.128	5.25e−05***	7.61E−05
T cell exhaustion	PD-1 (PDCD1)	0.245	1.56e−16***	3.77E−16	0.182	7.80e−09***	1.74E−08
	CTLA4	0.344	5.46e−32***	5.28E−31	0.299	6.23e−22***	6.02E−21
	LAG3	0.297	7.47e−24***	3.61E−23	0.26	8.25e−17	4.79E−16
	TIM-3 (HAVCR2)	0.248	8.13e−17***	2.20E−16	0.212	1.43e−11***	3.77E−11
	GZMB	0.361	3.63e−35***	5.26E−34	0.311	1.00e−23***	1.45E−22

*BRCA* breast invasive carcinoma, *TAM* tumor-correlated macrophage, *Tfh* follicular helper T cell, *Th* T helper cell, *Treg* regulatory T cell.

Cor, R value of Spearman’s correlation; None, correlation without adjustment. Purity, correlation adjusted by purity. *P < 0.01 (1e−02); **P < 0.001 (1e−03); ***P < 0.0001 (1e−04).

### The relationship between GSDMC and immune checkpoints in BRCA

SIGLEC15, PDCD1LG2 (PD-L2), TIGIT, PDCD1 (PD-1), CD274 (PD-L1), CTLA4, LAG3, and HAVCR2 (TIM3) are transcripts related to immunological checkpoints that perform a vital function in tumor immune evasion. Taking into account that GSDMC might be the potential oncogene in BRCA, the relationship of GSDMC with PDCD1LG2, SIGLEC15, LAG3, TIGIT, CTLA4, CD274, PDCD1, and HAVCR2 were assessed. As a result, we found that the expression levels of GSDMC were a significant positive correlation with PDCD1LG2, TIGIT, LAG3, CD274, CTLA4, HAVCR2, and PDCD1 in BRCA (Fig. 13B). On the contrary, GSDMC expression was a significant positive correlation with SIGLEC15 in BRCA (Fig. 13B). These findings indicated that tumor immune evasion and antitumor immunity might be implicated in GSDMC facilitated carcinogenic processes of BRCA.

### Alterations and mutations in GSDMC and its frequently altered neighbor genes in BRCA

**Figure 14:**
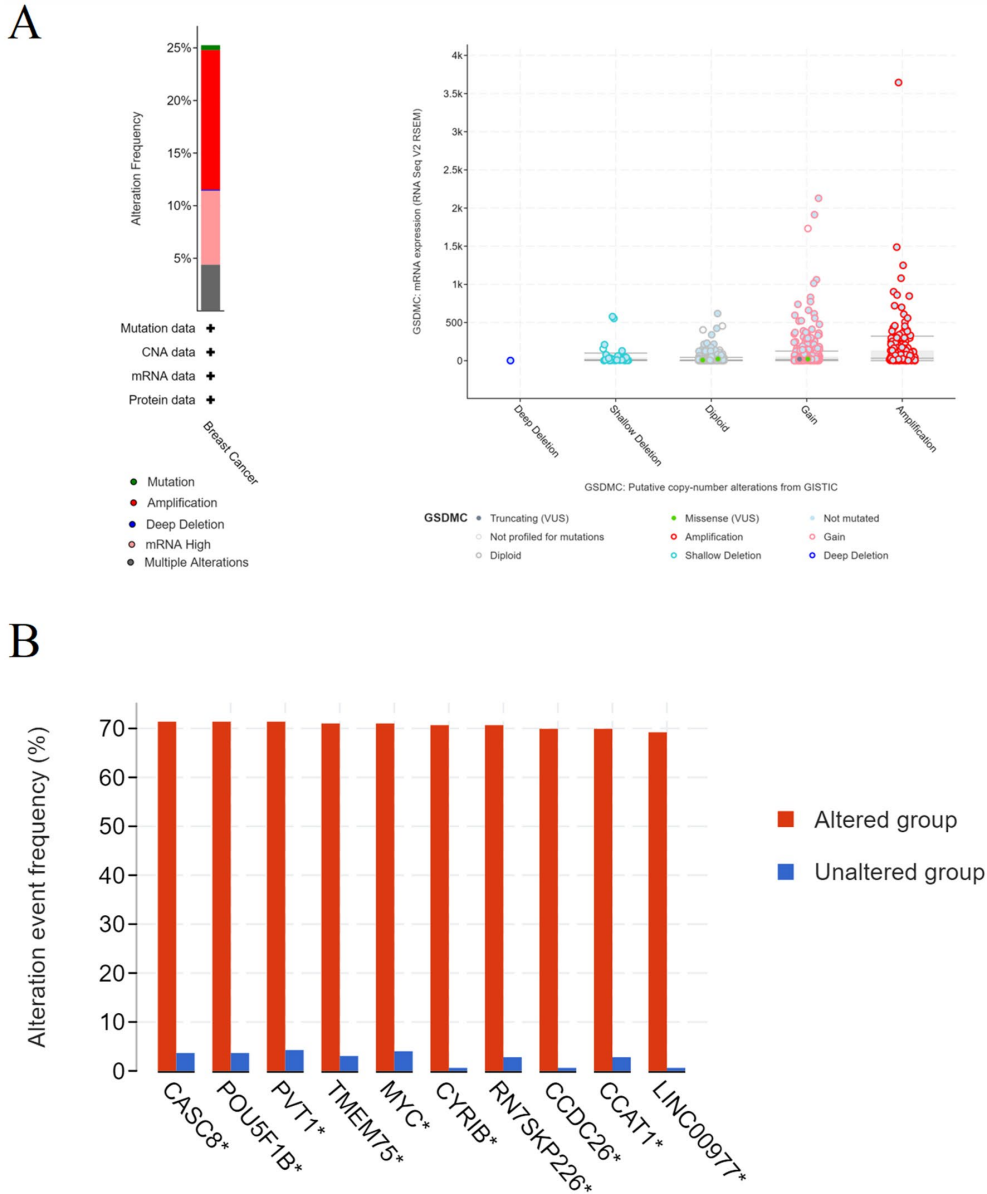
(**A**) GSDMC alterations in BRCA; (**B**) the 10 most frequently altered neighbor genes for GSDMC in BRCA (cBioPortal). *P < 0.05, **P < 0.01, ***P < 0.001.

We utilized the cBioPortal online tool to assess GSDMC alterations in the BRCA dataset (TCGA, Firehose Legacy). GSDMC altered in 25% of 1108 patients with BRCA (Fig. 14A). We also calculated putative copy-number alterations (CNA) and mutations from GISTIC of GSDMC in BRCA (Fig. 14A). Next, we constructed the 10 most commonly modified neighbor genes for GSDMC in BRCA (Fig. 14B). The findings illustrated that GSDMC alterations were considerably correlated with the cancer-related genes in BRCA, including CASC8, POU5F1B, PVT1, TMEM75, MYC, CYRIB, RN7SKP226, CCDC26, CCAT1, and LINC00977 (Fig. 14B).

## Discussion

Breast cancer is the considered highest incident cancer type and the first contributor to cancer-associated fatality in women worldwide^10,34^. Based on molecular phenotyping and genotyping, targeted therapies and immunotherapy of BC have rapidly evolved in recent years^1,5,9^. However, mortality rates of BRCA remain high and treatments are limited^2,35^.

Early studies have demonstrated that pyroptosis is correlated to tumors^14,36^. Pyroptosis can enhance antitumor immunity for its immunogenic nature^37^. Recently research demonstrates pyroptosis of tumor cells may overcome tumor cells’ apoptosis resistance and promotes antitumor immunity^38,39^. However, the specific mechanism of tumors pyroptosis remains poorly understood.

GSDMC, as an affiliate of the GSDM family, is mainly expressed in the skin, spleen, trachea, intestines, bladder, and gastrointestinal^26,28,40^. Some studies suggest that GSDMC expression level is correlated with some tumors, such as lung adenocarcinoma, metastatic melanoma, esophageal cancer, and gastric cancer^29,40–42^. The latest study indicates that GSDMC and PD-L1 can lead to necrosis of breast cancer tissue by switching apoptosis to pyroptosis in the hypoxic area^12^. But the molecular mechanism of GSDMC in tumors is poorly understood.

In this research, we assessed the mRNA expression levels of GSDMC in pan-tumors and matching non-cancer normal tissues utilizing TGGA and TIMER databases. Taken together, upregulation of GSDMC was found in UCEC, BRCA, READ, CHOL, LUSC, COAD, LUAD, KICH, LIHC, and KIRC. These data together with some studies mentioned above suggested that GSDMC might serve as the pivotal player in the carcinogenesis of these kinds of cancer^27,28,40^. Next, we carried out a pan-cancer survival analysis of the expression of GSDMC utilizing the TCGA database, after which the GEPIA database and KM plotter were employed for validation. Finally, a combination of the expression and prognostic significance of GSDMC in several kinds of cancers, we found that elevated expression of GSDMC might play as an unfavorable prognostic biomarker in BRCA patients. IHC analysis of 15 pairs of BC tumor tissues and corresponding adjacent normal tissues also revealed significantly higher expression of GSDMC in BC tissues than normal tissues. Furthermore, GSDMC expression and prognosis analysis of BC in GEO database showed the same results. Thus, further experimental validation is needed to investigate the link between the expression of GSDMC and the prognosis of cancer patients in BC and other kinds of cancers, including PAAD, COAD, KICH, etc.

It has been well documented that ceRNA theory explains interactions among mRNA and ncRNAs (miRNAs, lncRNAs, and circular RNAs (circRNAs))^32,43^. LncRNAs affects the miRNA affinity of target mRNA by attaching to similar miRNA response elements, thereby regulating gene expression at the transcriptional level^31,33^. We predicted potential miRNA candidates of GSDMC via several target gene prediction websites, consisting of miRDB, miRmap, TargetScan, miRcode, miRWalk, and DIANA-microT, and finally found 15 miRNAs. We found that GSDMC was only significantly negatively associated with hsa-miR-573 (MIR573) and positively linked to hsa-miR-548ao-5p (MIR548AO) in BRCA (*P* < 0.001). Meanwhile, no statistical expression link was observed between GSDMC and other miRNAs. The observed findings also illustrated that the levels of hsa-miR-573 expression were lower than normal tissue control in BRCA. Next, our survival analysis showed that hsa-miR-573 acting as tumor-suppressive miRNAs in BRCA. Taking together correlation analysis, expression analysis, and survival analysis, we suggested that hsa-miR-573 might serve as the most potential regulatory miRNA of GSDMC in BRCA. Early research also showed that hsa-miR-573 was a negative regulator of cell proliferation, migration, and invasion of pancreatic cancer^44,45^.

**Figure 15:**
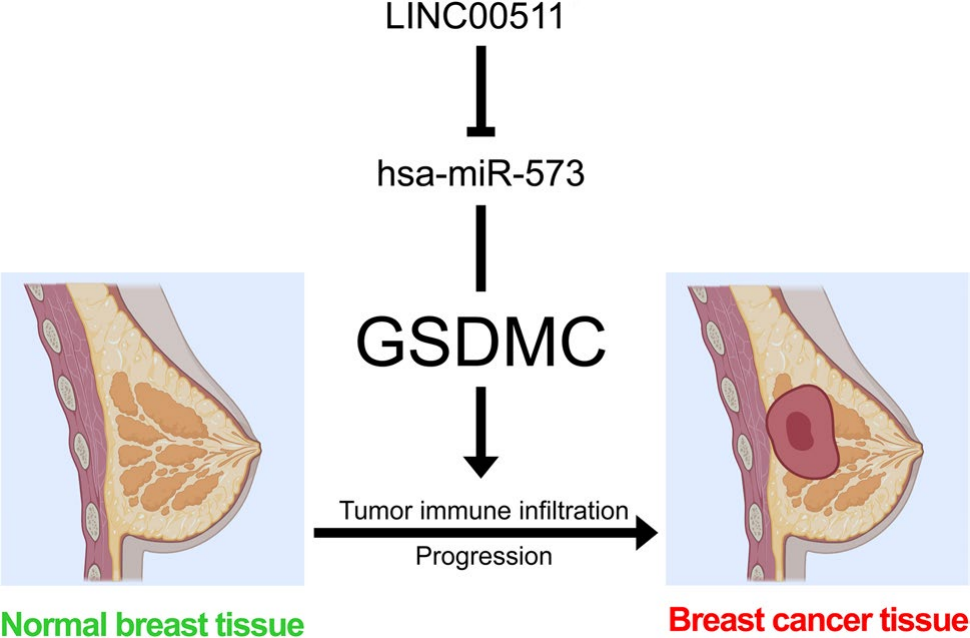
The model of LINC00511/GSDMC/hsa-miR-573 axis in carcinogenesis of BRCA.

Then we selected 39 potential candidate lncRNAs that interacted with hsa-miR-573 by using DIANA-LncBase v.2. Based on ceRNA hypothesis proposes, there ought to be a positive relationship between potential lncRNA and GSDMC and negative relationship between potential lncRNA and hsa-miR-573, and it should be oncogenic lncRNAs in BRCA. By correlation analysis, survival analysis, and expression analysis, LINC00511 was chosen as the key potential upstream lncRNA of GSDMC/hsa-miR-573 axis in BRCA. In early studies, LINC00511 was determined as oncogenic lncRNAs in several tumors, including gastric cancer, lung squamous cell carcinoma, cervical cancer, bladder carcinoma, glioma, and breast cancer^46–50^. Taken together, LINC00511/hsa-miR-573/GSDMC axis was well identified as potential regulatory pathways in BRCA (Fig. 15).

It is well known that breast cancer is a highly clinical and molecular heterogeneous disease. For this reason, we performed prognostic analysis of GSDMC, hsa-miR-573 and LINC00511 in patients with differently molecular subtyping of BRCA. The results indicated that LINC00511/hsa-miR-573/GSDMC axis might act in luminal B BRCA. Additional research would be necessary.

As an affiliate of the Gasdermin superfamily, GSDMC participated in the modulation of epithelial cell immune-related activities. Therefore, for another crucial facet of this study, we explored the link between GSDMC and tumor immune infiltration in BRCA. The tumor immune cell infiltration is an indispensable component of the tumor immune microenvironment which is closely associated with tumor progression, clinical outcomes as well as immunotherapy responses^51,52^. Our research showed a considerable correlation between levels of GSDMC expression and immune cell infiltration of myeloid dendritic cells, CD4+ T cells, CD8+ T cells, and neutrophils in BRCA. Moreover, we discovered considerable positive associations between GSDMC expression and several immunological biomarkers of these infiltrated immune cells. Some reports also found that GSDMC performed an instrumental function in adaptive immune responses^30^. These results indicated that GSDMC assumed a vital function in modulating immune cell infiltration in BRCA, with specifically powerful influence on CD4+ T cells, CD8+ T cells, myeloid dendritic cells, and neutrophils.

Immunological and T-cell-infiltrated tumors respond favorably to inhibition of immunological checkpoints, and anti-tumor immune response may be amplified through blocking of immune checkpoints^53,54^. SIGLEC15, PDCD1LG2 (PD-L2), TIGIT, PDCD1 (PD-1), HAVCR2 (TIM3), CTLA4, LAG3, and CD274 (PD-L1) are transcripts related to immunological checkpoints that have a function in tumor immune evasion. As a result, we found that, besides SIGLEC15, the levels of GSDMC expression were considerably positively associated with the 5 immune checkpoints in BRCA. Hou et al. identified a non-immunological checkpoint role of PD-L1 and found GSDMC/caspase-8 cause tumour necrosis via mediate a non-canonical pyroptosis pathway in cancer cells. Our research could provide more clues for GSDMC/PD-L1 therapeutic strategies. All these outcomes illustrated that tumor immune evasion and antitumor immunity might be implicated in GSDMC mediated carcinogenic processes of BRCA.

Besides, we explored alterations and mutations in GSDMC and its frequently altered neighbor genes in BRCA, then found that GSDMC alterations were considerably correlated with the cancer-associated genes in BRCA, including CASC8, POU5F1B, PVT1, TMEM75, MYC, CYRIB, RN7SKP226, CCDC26, CCAT1 and LINC00977.

In summary, we discovered that elevated GSDMC expression was considerably linked to a worse prognosis in BRCA. Next, we identified a LINC00511/hsa-miR-573/GSDMC axis as potential regulatory pathways in BRCA. For another important aspect of this research, our research demonstrated that GSDMC was a pivotal player in BC carcinogenesis via elevating tumor immune cell infiltration and the expression of immunological checkpoints. It is expected to provide new ideas for the link between pyroptosis and tumor immunotherapy. Nonetheless, this process requires much more fundamental research and extensive clinical trials.

## Materials and methods

### Data processing and differential expression analysis, survival analysis and correlation analysis

The UCSC Xena dataset was used to acquire TCGA and GTEx expression and clinical information (https://toil-xena-hub.s3.us-east-1.amazonaws.com/download/TcgaTargetGtex_rsem_gene_tpm.gz; Full metadata)^55^. Dataset ID: TcgaTargetGtex_rsem_gene_tpm. Raw counts of RNA-sequencing data (level 3) and matching clinical data contains 10,363 tumor tissues and 730 adjacent tissues from 18 types of cancer. In BRCA, we obtained 1109 breast cancer tissues and 113 adjoining tissues. Three independent BRCA gene expression profiles (GSE29431, GSE31448 and GSE42568) were downloaded from the Gene Expression Omnibus (GEO) database (https://www.ncbi.nlm.nih.gov/geo/) and processed for analysis^56^. Detailed information of datasets was listed in Table S1. All analytical methods were carried out utilizing the R software version v4.0.3. Expression analysis and Survival curves were drawn using the R packages “ggplot2”, “survival”, and “survminer”. The Log-rank tests as well as the univariate Cox proportional hazards regression generated hazard ratio (HR) and P-values with a confidence interval (CI) of 95% in KM curves. The R package “ggstatsplot” was used to analyze two-gene correlations. To examine the link between quantitative variables, Spearman’s correlation or Pearson correlation analysis was utilized.

### Tissue samples

15 paired breast cancer tissues and adjacent normal tissues were collected from Liuzhou People’s Hospital. A total of 15 patients had undergone surgery, and received chemoradiotherapy or induction chemotherapy. Relevant clinical data was shown in Table S2. This study was approved by the Ethics Committee of Liuzhou People’s Hospital (Reference No. KY2021-021-01), and was performed according to the Declaration of Helsinki. All tissues were pathologically examined.

### Immunohistochemistry (IHC) analysis

Formalin fixed paraffin-embedded tissues (4 µm thick) were analyzed by IHC with GSDMC antibody (1:50; Affinity, China) and horseradish peroxidase conjugated secondary antibodies (Maxim, China). For IHC quantification, the sections were analyzed using DM2000 LED microscope (Leica, Germany) and three randomly selected areas were photographed. The diagnoses were confirmed by three pathology specialists. The integral optical density (IOD) was determined by the Image-Pro Plus 6.0 software (Media Cybernetics, USA).

### TIMER database analysis

TIMER (https://cistrome.shinyapps.io/timer/) dataset comprise six tumor-infiltrating immune subsets^57^. The levels of six subsets are precalculated for 10,897 tumors in 32 kinds of cancer from the TCGA. The database analyzed gene expression and tumor immune infiltration (Dendritic cells, Macrophages, Neutrophils, B cells, CD4+ T cells, and CD8+ T cells) in a variety of cancers. We examined the mRNA expression of GSDMC in pan-cancer patients utilizing the TIMER dataset. Subsequently, we investigated the expression association between GSDMC and specific immune infiltrating cell subsets markers.

### GEPIA2 database analysis

Gene Expression Profiling Interactive Analysis (GEPIA) contains the RNA sequence expression information of 9736 tumors and 8587 non-tumor normal specimens from the TCGA and GTEx projects, which is used to analyze its standard processing pipelines^58^. GEPIA2 (http://gepia2.cancer-pku.cn/) is an updated version of GEPIA. We used GEPIA2 to determine the connection between the mRNA expression of GSDMC and patient prognosis in pan-cancers. We also examined the expression of LINC00511 and hsa-miR-573 in BRCA and adjoining tissues and the link between the levels of mRNA expression of hsa-miR-573 and LINC00511 and BRCA patient prognosis.

### Kaplan–Meier plotter analysis

Kaplan–Meier plotter (KM plotter; http://kmplot.com/analysis/) is a commonly used online repository that explores the influence of multiple genes on the prognosis of 21 distinct kinds of cancers in a huge sample size. KM plotter contains survival data and microarray gene expression information derived from the TCGA, Gene Expression Omnibus (GEO), and European Genome-Phenome Archive^59^. We investigated the predictive values of GSDMC in pan-cancers utilizing KM plotter. We also analyze the prognostic values of hsa-miR-573 and LINC00511 in BRCA patients.

### Candidate miRNA prediction

We predicted potential miRNA candidates of GSDMC via various target gene forecasting website, comprising of miRDB (http://mirdb.org/)^60^, miRmap (https://mirmap.ezlab.org/app/)^61^, TargetScan (http://www.targetscan.org/vert_71/)^62^, miRcode (http://www.mircode.org/index.php?gene=gsdmc&mirfam=&class=&cons=&trregion=), miRWalk (http://zmf.umm.uni-heidelberg.de/apps/zmf/mirwalk2/generetsys-self.html)^63^ and DIANA-microT (http://diana.imis.athena-innovation.gr/DianaTools/index.php?r=microT_CDS/results&keywords=ENSG00000147697&genes=ENSG00000147697%20&mirnas=&descr=&threshold=0.7)^64^. For subsequent analyses, we chose miRNAs candidates which, as indicated above, were often found in over three systems. These projected miRNAs were therefore chosen as GSDMC miRNAs candidates.

### DIANA-LncBase v2 database analysis

DIANA-LncBase v2 database (http://carolina.imis.athena-innovation.gr/diana_tools/web/index.php?r=lncbasev2%2Findex-predicted) is a database for assessing the link between lncRNAs and miRNAs^65^. LncBase Predicted v.2 was introduced to predicted potential candidate lncRNAs that interact with hsa-miR-573 in breast tissues and mammary gland tissues (Threshold > 7).

### Immune cell infiltration and immune checkpoints analysis

The TCGA database was utilized to retrieve raw counts of RNA-sequencing data (level 3) in which contains 1109 BC tissues and 113 adjoining tissues. We investigated immune cell infiltration and immunological checkpoints of GSDMC in BRCA utilizing R packages “immunedeconv”, “ggplot2”, “pheatmap”, and “ggstatsplot” to produce accurate immune infiltration estimates. R foundation for statistical computation (2020) version 4.0.3 was utilized to implement all of the aforementioned analytic techniques.

### TCGA data and cBioPortal analysis

The cBioPortal for Cancer Genomics supports analysis, visualization, as well as downloading of cancer genomics datasets (http://www.cbioportal.org/)^66^. The BRCA dataset (TCGA, Firehose Legacy) which contains data of 1108 BRCA patients, was selected for GSDMC analysis via cBioPortal database. The genomic signatures comprised of mutations, putative copy-number alterations (CNA), mRNA expression z-scores (RNA Seq V2 RSEM), and protein expression Z-scores (RPPA) from GISTIC. The computation of co-expression was carried out as per the online instructions of cBioPortal.

### Statistical analysis

We analyzed data by a log-rank test, such as fold-change, Hazard ratio (HR), and P*-*values. We measured the extent of correlation between particular variables via Spearman’s correlation analysis or Pearson correlation analysis, with the r values to measures the relationship strength. P-Value or Log-rank P-value of < 0.05 was judged as having statistical significance.

### Ethics approval and consent to participate

This study was approved by the Ethics Committee of Liuzhou People’s Hospital (Reference No. KY2021-021-01), and was performed according to the Declaration of Helsinki. Written informed consent forms were obtained from all subjects.

## Supplementary Information

Supplementary Legends.

Supplementary Figure S1.

Supplementary Figure S2.

Supplementary Figure S3.

Supplementary Figure S4.

Supplementary Table S1.

Supplementary Table S2.

## Data Availability

The UCSC Xena dataset was used to acquire TCGA and GTEx expression and clinical information (https://toil-xena-hub.s3.us-east-1.amazonaws.com/download/TcgaTargetGtex_rsem_gene_tpm.gz; Full metadata). Dataset ID: TcgaTargetGtex_rsem_gene_tpm. Raw counts of RNA-sequencing data (level 3) and matching clinical data contains 10,363 tumor tissues and 730 adjacent tissues from 18 types of cancer. All the datasets were retrieved from the publishing literature, so it was confirmed that all written informed consent was obtained.

## Abbreviations

BC: Breast cancer
GSDMC: Gasdermin C
TCGA: The Cancer Genome Atlas
ncRNAs: Noncoding RNAs
IHC: Immunohistochemistry
GSDM: Gasdermin
UV: Ultraviolet
miRNAs: MicroRNAs
lncRNAs: Long noncoding RNAs
CI: Confidence interval
TIMER: Tumor Immune Estimation Resource
GEPIA: Gene Expression Profiling Interactive Analysis
KM plotter: Kaplan–Meier plotter
HNSC: Head and neck squamous cell carcinoma
KIRC: Kidney renal clear cell carcinoma
BC: Breast cancer
RNA Seq V2 RSEM: MRNA expression z-scores
RPPA: Protein expression Z-scores
CNA: Copy-number alterations
ECL: Extracellular loop
EDG: Endothelial differentiation gene
GEO: Gene Expression Omnibus
GEPIA: Gene Expression Profiling Interactive Analysis
GPCRs: G-protein coupled receptors
HR: Hazard ratio
BLCA: Bladder urothelial carcinoma
BRCA: Breast invasive carcinoma
KICH: Kidney chromophobe
KIRP: Kidney renal papillary cell carcinoma
LIHC: Liver hepatocellular carcinoma
ESCA: Esophageal carcinoma
LUAD: Lung adenocarcinoma
LUSC: Lung squamous cell carcinoma
PRAD: Prostate adenocarcinoma
READ: Rectum adenocarcinoma
UCEC: Uterine corpus endometrial carcinoma
CHOL: Cholangial carcinoma
COAD: Colon adenocarcinoma
CECS: Cervical squamous cell carcinoma and endocervical adenocarcinoma
STAD: Stomach adenocarcinoma
DLBC: Lymphoid Neoplasm Diffuse Large B-cell Lymphoma
THCA: Thyroid carcinoma
GBM: Glioblastoma multiforme
PAAD: Pancreatic adenocarcinoma
THYM: Thymoma
ACC: Adrenocortical carcinoma
BLCA: Bladder urothelial carcinoma
PCPG: Pheochromocytoma and Paraganglioma
UCEC: Uterine corpus endometrial carcinoma
UCS: Uterine Carcinosarcoma
OS: Overall survival
DFS: Disease-free survival
RFS: Relapse-free survival
PPS: Post-progression survival
DSS: Disease-specific survival
DMFS: Distant metastasis-free survival
PFI: Progress free interval
FP: First progression
TAM: Tumor-associated macrophages
NK cells: Natural killer cells
MDCs: Myeloid dendritic cells
Tfh: Follicular helper T cell
Th: T helper cell
CircRNAs: Circular RNAs
