# Supplementary material for: LINC00511/hsa-miR-573 axis-mediated high expression of Gasdermin C associates with dismal prognosis and tumor immune infiltration of breast cancer
Source: Sci Rep. 2022 Aug 30;12:14788. doi: 10.1038/s41598-022-19247-9 (PMC9428000; doi:10.1038/s41598-022-19247-9)
Supplement: Supplementary file 1 — Supplementary Legends. [file 41598_2022_19247_MOESM1_ESM.docx]

**Figure S1.** Prognostic analysis of GSDMC mRNA expression levels in different tumor types in the TCGA database(A-AI)

**P* < 0.05, ***P* < 0.01, ****P* < 0.001

Abbreviations: OS, overall survival; DSS, disease-specific survival.

**Figure S2**. Prognostic analysis of GSDMC mRNA expression levels in various human cancers determined by GEPIA database (A-BL)

Abbreviations: OS, overall survival; DFS, disease-free survival.

**Figure S3.** Kaplan-Meier survival curves comparing the high and low expression of GSDMC in different types of cancers in the Kaplan-Meier plotter databases. (A-AL)

Abbreviations: OS, overall survival; DFS, disease-free survival; RFS, relapse-free survival; PPS, post-progression survival; DMFS, distant metastasis-free survival.

**Figure S4. A:** Association between GSDMC protein expression and survival prognosis of 15 BC tissues. **B:** Relative mRNA expression of LINC00511 in BC and paired normal tissues from GEO database. (In GSE29431) C: Kaplan-Meier survival curves comparing the high and low expression of LINC00511 in BC from GEO database (In GSE42568).

Abbreviations: BC, breast cancer; PFS: progression-free survival.
