# Supplementary figures and images for: LINC00511/hsa-miR-573 axis-mediated high expression of Gasdermin C associates with dismal prognosis and tumor immune infiltration of breast cancer
Source: Sci Rep. 2022 Aug 30;12:14788. doi: 10.1038/s41598-022-19247-9 (PMC9428000; doi:10.1038/s41598-022-19247-9)

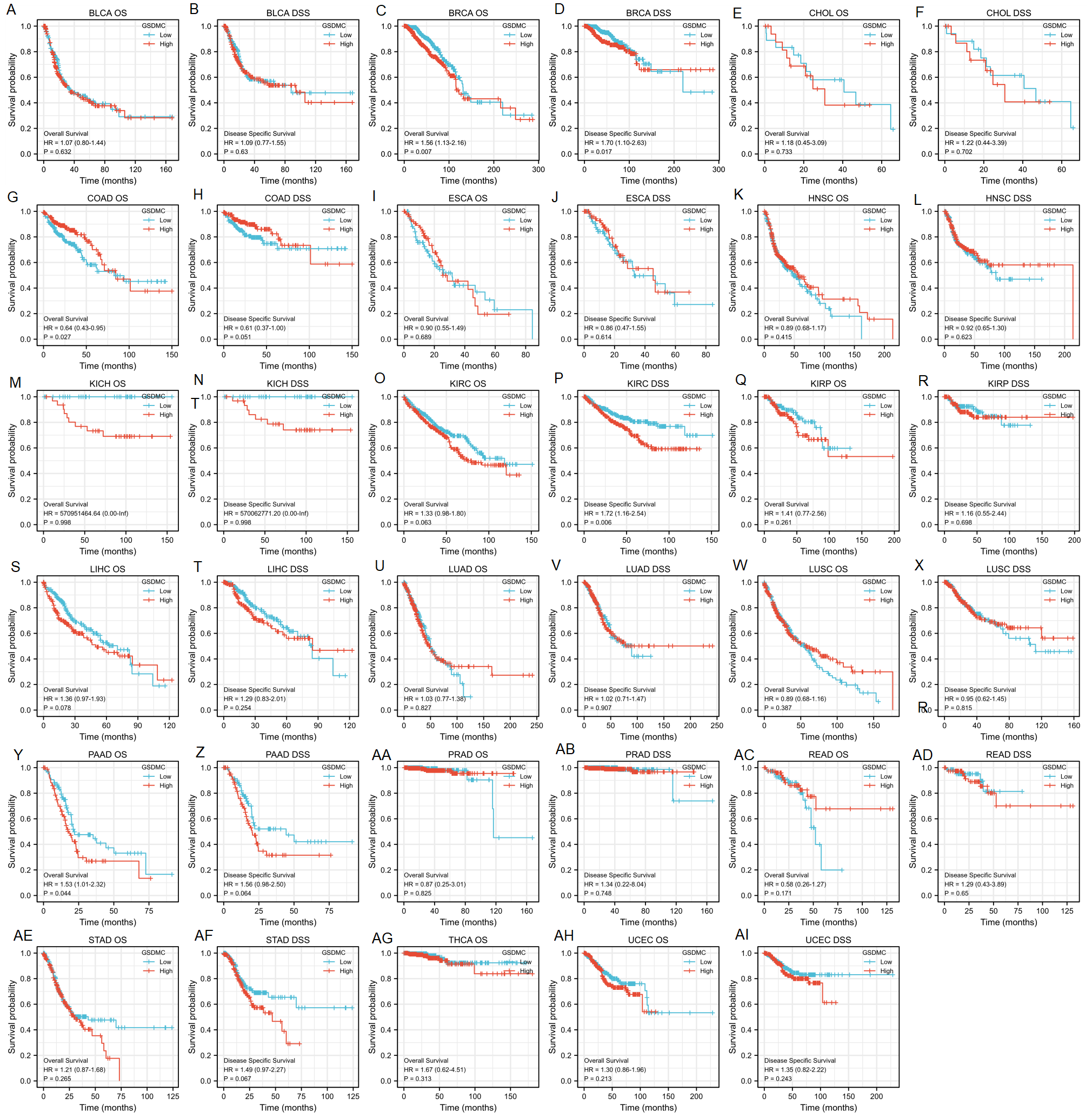

Supplement: Supplementary file 2 — Supplementary Figure S1. [file 41598_2022_19247_MOESM2_ESM.tif]

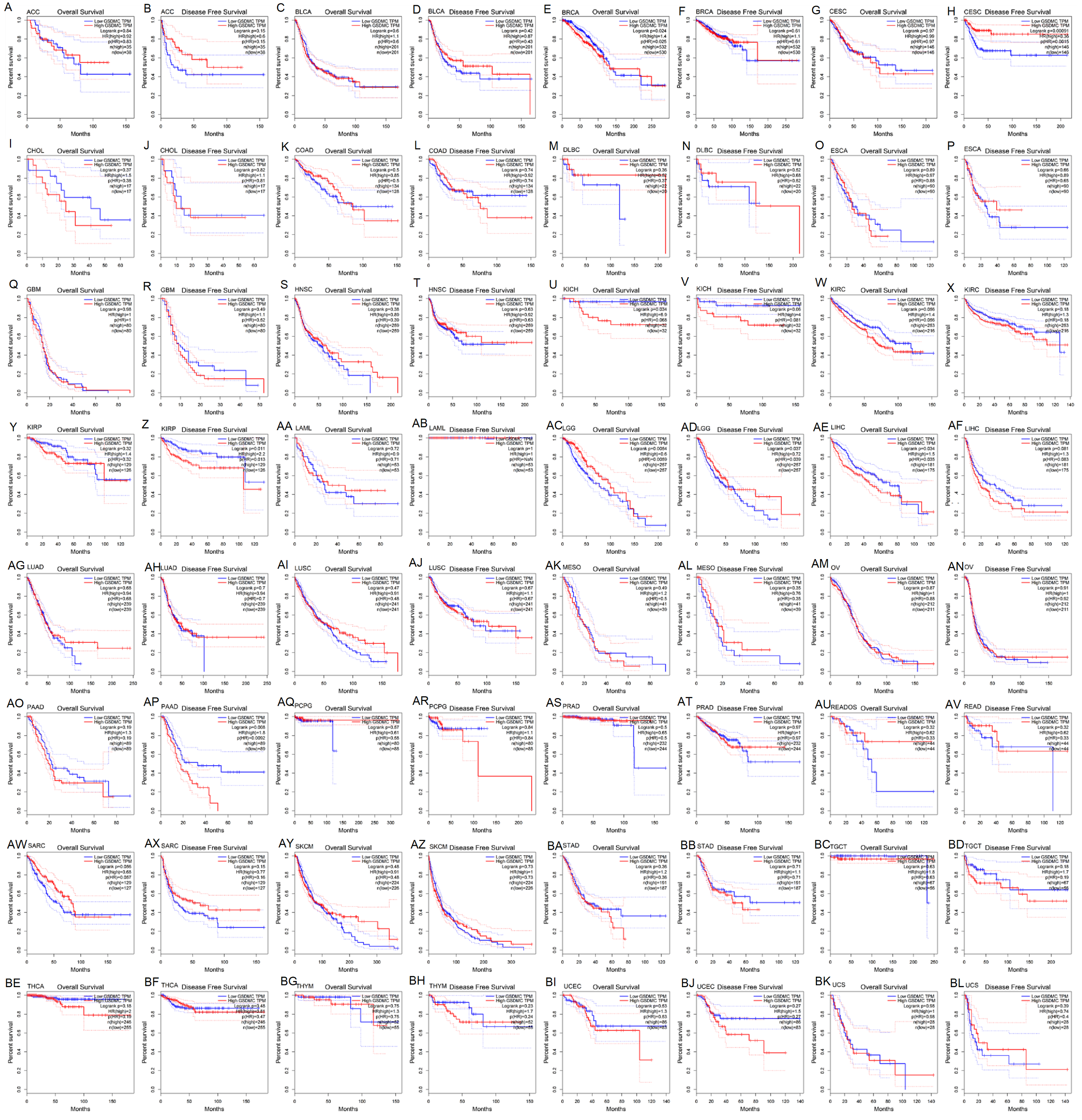

Supplement: Supplementary file 3 — Supplementary Figure S2. [file 41598_2022_19247_MOESM3_ESM.tif]

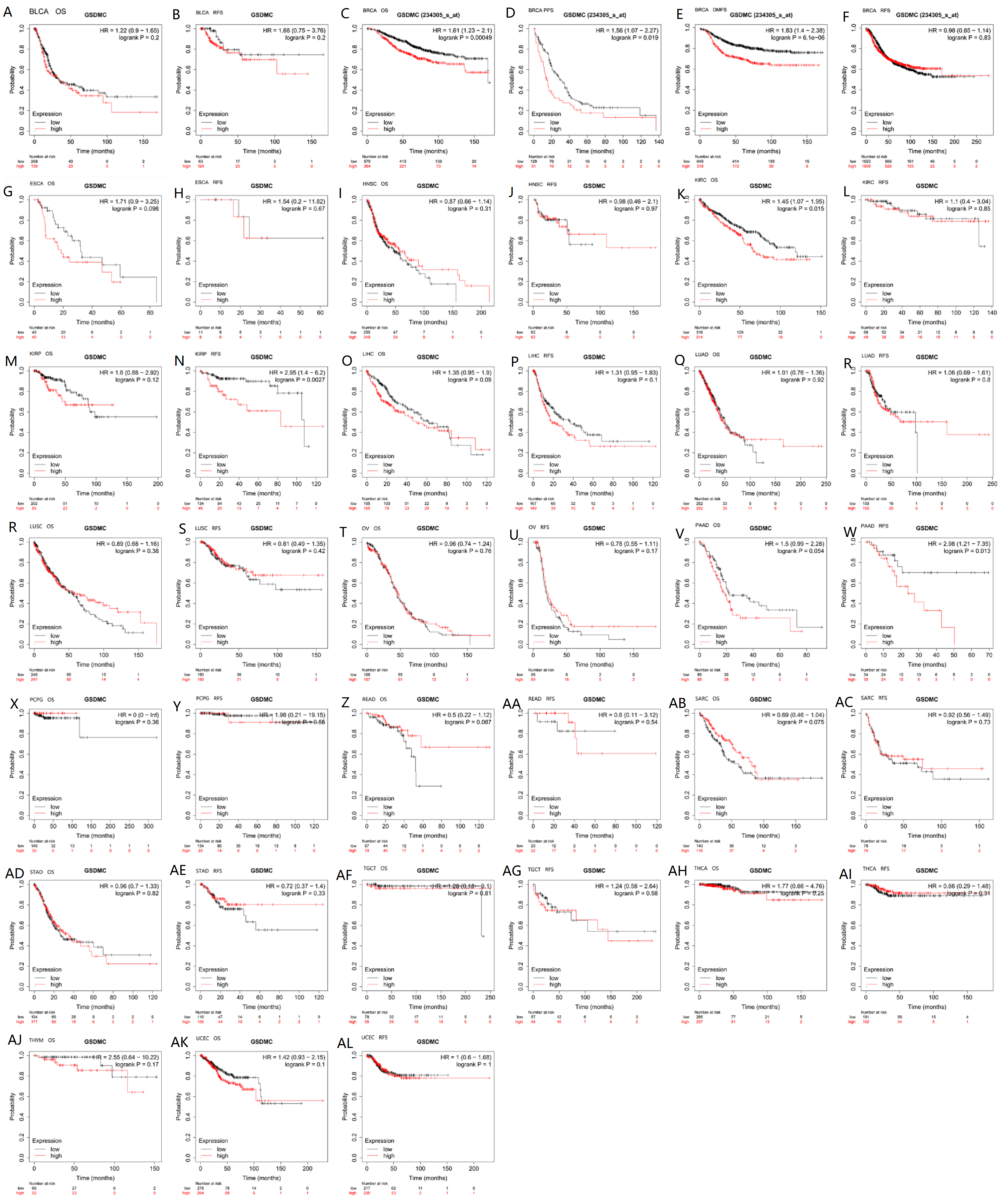

Supplement: Supplementary file 4 — Supplementary Figure S3. [file 41598_2022_19247_MOESM4_ESM.tif]

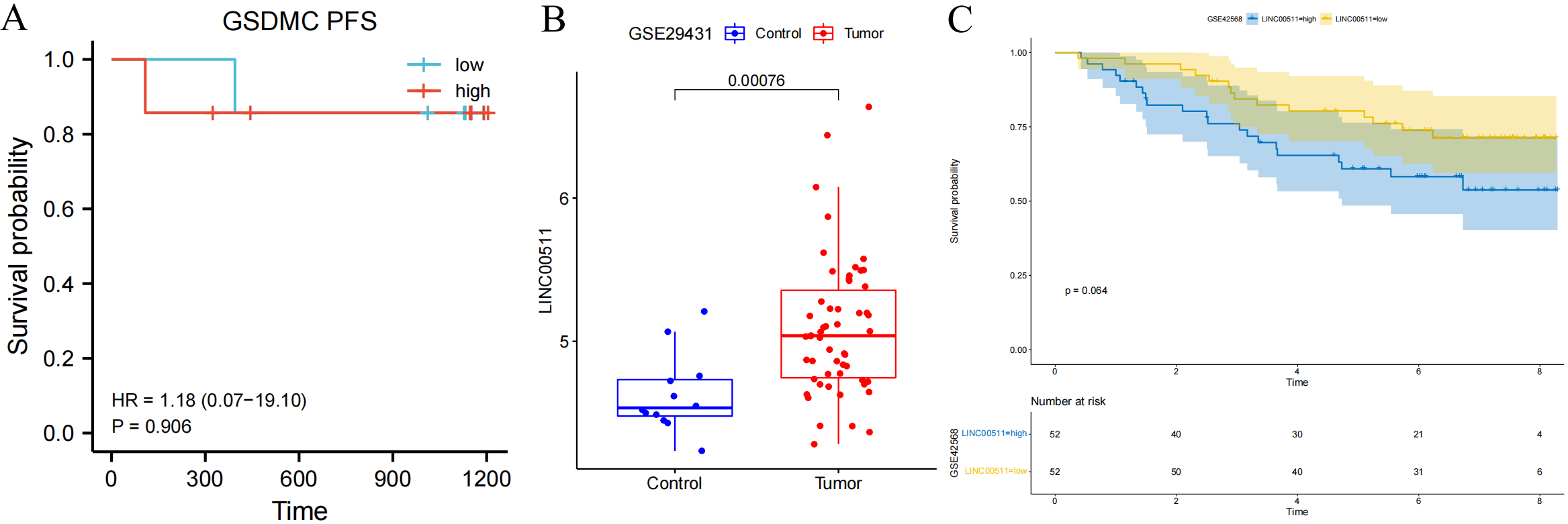

Supplement: Supplementary file 5 — Supplementary Figure S4. [file 41598_2022_19247_MOESM5_ESM.tif]
