# Supplementary material for: LINC00511/hsa-miR-573 axis-mediated high expression of Gasdermin C associates with dismal prognosis and tumor immune infiltration of breast cancer
Source: Sci Rep. 2022 Aug 30;12:14788. doi: 10.1038/s41598-022-19247-9 (PMC9428000; doi:10.1038/s41598-022-19247-9)
Supplement: Supplementary file 6 — Supplementary Table S1. [file 41598_2022_19247_MOESM6_ESM.docx]

| Datasets | Contributor | Disease type | Experimental platform | Number of cases (cancer/control） |
| --- | --- | --- | --- | --- |
| GSE29431 | Cuadros M, Cano C, et al. (2019) | Breast cancer | Affymetrix Human Genome U133 Plus 2.0 Array | 54/12 |
| GSE31448 | Sabatier R, Finetti P, et al. (2019) | Breast cancer | Affymetrix Human Genome U133 Plus 2.0 Array | 263/31 |
| GSE42568 | Clarke C, Madden SF, et al.（2013） | Breast cancer | Affymetrix Human Genome U133 Plus 2.0 Array | 104/17 |

**Table S1 Information of the Selected GEO Datasets**
