# Supplementary material for: LINC00511/hsa-miR-573 axis-mediated high expression of Gasdermin C associates with dismal prognosis and tumor immune infiltration of breast cancer
Source: Sci Rep. 2022 Aug 30;12:14788. doi: 10.1038/s41598-022-19247-9 (PMC9428000; doi:10.1038/s41598-022-19247-9)
Supplement: Supplementary file 7 — Supplementary Table S2. [file 41598_2022_19247_MOESM7_ESM.docx]

**Table S2.** Clinical characteristics of patients with BC.

| Characteristic | levels | Overall |
| --- | --- | --- |
| n |  | 15 |
| Age | ≦50 | 7 (46.7%) |
|  | >50 | 8 (53.3%) |
| Menopausal status | Premenopausal | 7 (46.7%) |
|  | Postmenopausal | 8 (53.3%) |
| Tumor size | ≦2cm | 6 (40.0%) |
|  | >2cm | 9 (60.0%) |
| Lymph node metastasis | Yes | 6 (40.0%) |
|  | No | 9 (60.0%) |
| Histological grade | I-II | 10 (66.7%) |
|  | III | 5 (33.3%) |
| ER status | Negative | 5 (33.3%) |
|  | Positive | 10 (66.7%) |
| PR status | Negative | 6 (40.0%) |
|  | Positive | 9 (60.0%) |
| HER-2/neu status | Negative | 6 (40.0%) |
|  | Positive | 9 (60.0%) |
| TNM stage | I-II | 9 (60.0%) |
|  | III-IV | 6 (40.0%) |
| Molecular subtype | LuminalA | 3 (20.0%) |
|  | Luminal B | 8 (53.3%) |
|  | HER2-enriched | 1 (6.7%) |
|  | Triple Negative | 3(20.0%) |

Abbreviations: BC, breast cancer.
